# Methanogenic symbionts of anaerobic ciliates are host and habitat specific

**DOI:** 10.1093/ismejo/wrae164

**Published:** 2024-08-20

**Authors:** Daniel Méndez-Sánchez, Anna Schrecengost, Johana Rotterová, Kateřina Koštířová, Roxanne A Beinart, Ivan Čepička

**Affiliations:** Department of Zoology, Faculty of Science, Charles University, Viničná 7, 128 00 Prague 2, Czech Republic; Graduate School of Oceanography, University of Rhode Island, Narragansett, RI 02882, United States; Department of Zoology, Faculty of Science, Charles University, Viničná 7, 128 00 Prague 2, Czech Republic; Graduate School of Oceanography, University of Rhode Island, Narragansett, RI 02882, United States; Department of Marine Sciences, University of Puerto Rico Mayagüez, Mayagüez, PR 00680, United States; Department of Zoology, Faculty of Science, Charles University, Viničná 7, 128 00 Prague 2, Czech Republic; Graduate School of Oceanography, University of Rhode Island, Narragansett, RI 02882, United States; Department of Zoology, Faculty of Science, Charles University, Viničná 7, 128 00 Prague 2, Czech Republic

**Keywords:** anaerobiosis, archaea, endosymbionts, methane, symbiosis, syntrophy, transmission mode

## Abstract

The association between anaerobic ciliates and methanogenic archaea has been recognized for over a century. Nevertheless, knowledge of these associations is limited to a few ciliate species, and so the identification of patterns of host–symbiont specificity has been largely speculative. In this study, we integrated microscopy and genetic identification to survey the methanogenic symbionts of 32 free-living anaerobic ciliate species, mainly from the order Metopida. Based on Sanger and Illumina sequencing of the 16S rRNA gene, our results show that a single methanogenic symbiont population, belonging to *Methanobacterium*, *Methanoregula*, or *Methanocorpusculum*, is dominant in each host strain. Moreover, the host’s taxonomy (genus and above) and environment (i.e. endobiotic, marine/brackish, or freshwater) are linked with the methanogen identity at the genus level, demonstrating a strong specificity and fidelity in the association. We also established cultures containing artificially co-occurring anaerobic ciliate species harboring different methanogenic symbionts. This revealed that the host–methanogen relationship is stable over short timescales in cultures without evidence of methanogenic symbiont exchanges, although our intraspecific survey indicated that metopids also tend to replace their methanogens over longer evolutionary timescales. Therefore, anaerobic ciliates have adapted a mixed transmission mode to maintain and replace their methanogenic symbionts, allowing them to thrive in oxygen-depleted environments.

## Introduction

Symbiotic relationships between unicellular eukaryotes, or protists, and archaea or bacteria are prevalent across the major supergroups of eukaryotes. However, little is known about the biology, ecology, physiology, and evolution of many of these complex interactions [1, 2]. Ciliates, a well-known group of protists, have independently established multiple associations with a wide range of eukaryotes [3, 4] and prokaryotes [1, 3, 5] during their evolutionary history. Although most ciliates are aerobic, most of their major lineages (classes) include free-living or endobiotic taxa that have secondarily transitioned to an anaerobic lifestyle [6]. These transmissions have involved the modification of their mitochondria into mitochondrion-related organelles (MROs) and the acquisition of archaeal and/or bacterial symbionts that facilitate their fermentative metabolism [6–13]. This is assumed to be a syntrophic partnership in which symbiotic prokaryotes, commonly methanogens or sulfate-reducing bacteria, utilize the fermentation end-products of the host’s MROs, mainly H_2_, during methanogenesis or sulfate reduction, yielding a higher energetic efficiency for the host and resulting in methane or hydrogen sulfide production, respectively [6, 9, 13–20]. In some anoxic environments, the methanogenic symbionts of protists can significantly contribute to biogenic methane production, which is a potent greenhouse gas [14, 21–26].

Though it is assumed that methanogens form partnerships with most, if not all, anaerobic ciliates, only a few known symbionts associated with a limited number of ciliate species have been characterized using autofluorescence, transmission electron microscopy (TEM), fluorescence in situ hybridization (FISH), marker gene sequencing, and/or genomic analysis. These ciliates belong to the classes Armophorea and Plagiopylea, the subclasses Trichostomatia and Haptoria (Litostomatea), the family Anaerocyclidiidae (subclass Scuticociliatia), and the karyorelictid genus *Parduczia* [6, 8, 12, 16, 17, 27–44]. However, methanogenic endosymbionts have been observed in many more host species, predominantly in the obligate anaerobic, free-living, and species-rich order Metopida (Armophorea), using the characteristic autofluorescence of the methanogen F420 coenzyme [45–53].

Existing evidence suggests that each ciliate species harbors a single methanogen population [11, 12, 28, 29, 38, 44]. Although it is generally assumed that the symbionts are transmitted vertically during host division [11, 12], this evidence is limited to two ciliate species [54, 55]. Additionally, host and methanogen phylogenies do not show co-diversification patterns [6, 11, 12, 30], which would be expected in the case of strict vertical transmission [56]. In contrast, the horizontal acquisition of methanogenic symbionts is also presumed, as experimentally demonstrated in a cultivated aposymbiotic strain of *Trimyema* (Plagiopylea) that was successfully reinfected with two strains of symbiotic *Methanobacterium* [57, 58]. Overall, understanding of the methanogen–ciliate association remains largely unresolved, confounding our ability to discern, over both ecological and evolutionary timescales, drivers of fidelity and specificity between the host and the symbiont, as well as the transmission modes and mechanisms of maintenance of the symbiont.

Here, we present a comprehensive evaluation of host–symbiont partnerships within and among phylogenetic clades of host taxa, in the context of habitat type and host phylogeny. We studied the archaeal diversity associated with 54 strains of 32 anaerobic ciliate species, mostly metopids isolated from soil, marine/brackish, and freshwater sediments, via microscopy and Sanger and Illumina amplicon sequencing. In addition, we surveyed artificial co-cultures of several ciliate species to determine the potential for symbiont exchange between co-occurring pairs of ciliates.

## Materials and methods

### Source of anaerobic ciliates

Fifty-four anaerobic ciliate strains from long-term stable polyxenic cultures were used in this study, 42 from freshwater sediments, 11 from brackish/marine sediments, and one from soil (Table S1). All cultures were maintained in 10 ml of culture medium, American Type Culture Collection (ATCC) #802 for freshwater or #1525 for brackish/marine species. The cultures were polyxenic containing ciliates and unidentified prokaryotes in the sediment at the bottom of the tube. The sediment (ca. 1 ml) was subcultured biweekly into a new tube with fresh medium. Tubes were tightly closed and kept in darkness at room temperature (24°C) [49, 59]. One culture contained three ciliate species, five had two species each, and the remaining 41 had a single ciliate species (Table S1). Each ciliate strain was identified using 18S rRNA gene sequences amplified from individual cells (see below) (Table S1). Thirty-two species were examined: 30 metopids (Armophorea, Metopida), one *Caenomorpha* (Armophorea, Armophorida), and one *Trimyema* (Plagiopylea, Plagiopylida).

#### Co-cultivation experiments

To test the potential for horizontal transmission of methanogenic symbionts between different ciliate species and the stability of the symbionts, pairs of ciliates from already established cultures were mixed and cultivated together by adding 1 ml of each culture into 9 ml of medium. These artificial co-cultures were subcultured every 1 or 2 weeks (as described above). Seven mixed cultures were successfully established (Table S1). The species from the cultures were randomly examined at various intervals (Tables S1, S2).

### Detection of methanogenic archaea through microscopy

Symbiont autofluorescence via methanogen-specific coenzyme F420 was determined in living and fixed cells for most of the ciliate strains using a Zeiss Axioskop 2 plus microscope (Carl Zeiss Microscopy) with a UV filter (Zeiss filter set 01) according to [53] or a confocal microscope Leica TCS SP8. Symbiont-targeted FISH was performed using Cy3-labeled probes: ARC915 for archaea and MG1200b for order Methanomicrobiales [60–62] (Supplementary methods). TEM was implemented to localize the putative methanogenic symbionts in the host cells of several strains (Supplementary methods).

### Molecular identification of the ciliates and their methanogenic symbionts

#### Cell picking, DNA extraction, and sample preparation for SSU rRNA gene sequencing

For each ciliate strain, including the artificially mixed cultures, ~15 cells were handpicked with glass micropipettes, washed by transferring several times through sterile media, and usually starved for 30 min or up to 27 h [27, 63, 64]. Cells were finally transferred into 30 μl of UV-treated DNA/RNA Shield (Zymo Research, Irvine, CA, USA) and frozen at −20°C. Replicates (additional pools of cells) were collected for several strains (Table S2). Additionally, for most of the cultures, ciliates were removed from 30 μl of the culture using sterile glass micropipettes to obtain the culture medium containing only the free-living prokaryotic community (hereinafter control medium), which was dispensed into a tube with UV-treated DNA/RNA Shield and frozen at −20°C (Supplementary methods). Ciliates from the artificially mixed cultures were examined after up to 13 months of cultivation (Table S2).

#### PCR amplification and sequencing of host and symbiont SSU rRNA genes

DNA from picked cells and control medium was extracted using the DNeasy Blood & Tissue kit (Qiagen, Hilden, Germany) following the manufacturer’s directions. The identity of the ciliates and their methanogenic symbionts was assessed via amplification and Sanger sequencing of the 18S and partial 16S rRNA genes, respectively, using the eukaryotic primers Euk A and Euk B [65] for the ciliates, and the archaeal primers Arc915F and ArcR1326 [12, 34] for the methanogens. In parallel and using the same isolated DNA from each sample, amplicons for Illumina sequencing were prepared using archaea-specific barcoded primers Arc915F and ArcR1326. To obtain enough DNA and reduce PCR bias, PCR was done in triplicate for each sample. PCR products were subsequently pooled and purified, and then sequenced on a MiSeq system (Illumina 2x250bp) (Supplementary methods).

#### Identification and taxonomic assignment of amplicon sequence variants

Demultiplexing of the raw paired-end 16S rRNA gene reads was performed with BBMap [66] and then reads were imported into QIIME2 [67]. 16S rRNA gene amplicon reads were initially processed using QIIME2 and the DADA2 plugin. Primer sequence trimming, quality trimming, sequence denoising, chimera filtering, singleton removal, and sequence dereplication were performed with DADA2 [68]. Because paired-end reads could not be merged due to the poor quality of the forward reads, only the reverse reads were utilized in our analysis. For the taxonomic assignment, we utilized a Naive Bayes classifier pre-trained on the SILVA 138 99% OTUs (i.e. operational taxonomic units) full-length database and the q2-feature-classifier plugin [69, 70].

Amplicon sequence variants (ASVs), whose relative abundance was not >4% in any single sample, were assumed to represent contaminants and discarded in subsequent analyses [71, 72]. The remaining ASVs that were taxonomically assigned to a methanogen group were named according to their assigned genus or otherwise lowest assigned taxonomic rank. Sample metadata information is available in Table S3. Count tables and taxonomic assignments of ASVs after filtering and renaming are available in Tables S4–S6. ASV relative abundance per sample was computed after these filtering steps and fractional abundance plots were created with the PHYLOSEQ package in R [73]. Symbiont ASV and Sanger sequences that were obtained from the same ciliate strain were aligned, and pairwise nucleotide sequence identity distance matrices were computed using Clustal Omega (Table S7) [74] in Geneious Prime 11.0.9 + 11.

### Phylogenetic analysis of host 18S and symbiont 16S rRNA gene sequences

A dataset containing 18S rRNA gene sequences of the 54 ciliate strains used in this study was created and aligned using MAFFT (G-INS-i algorithm) [75]. The alignments were manually trimmed to the primer regions, using AliView v1.28 [76] (Supplementary methods).

The 131 16S rRNA sequences of methanogenic symbionts obtained from Sanger sequencing were de-replicated with vsearch (—derep_fulllength). A total of 32 unique sequences were identified and aligned along with reference and outgroup sequences obtained from GenBank, using the SILVA SINA aligner 1.2.11 [77], and manually trimmed to the Arc915F/ArcR1326 primer region using AliView v1.28. In parallel, all the obtained 16S rRNA gene sequences of *Methanobacterium* (65), *Methanoregula* (56), and *Methanocorpusculum* (10) were separately aligned with the reference and outgroup sequences as above. Pairwise distance matrices (Tables S8 and S9) were computed using Clustal Omega [74] in Geneious Prime 11.0.9 + 11.

The 16S and 18S rRNA gene phylogenetic trees were generated using the maximum likelihood method in RAxML 8.2.12 under the GTRGAMMAI model with 1000 bootstrap pseudoreplicates [78] in the web server The CIPRES Science Gateway V. 3.3 (https://www.phylo.org).

We conducted a full and partial Mantel test with the Vegan and NCF packages in R [79, 80] to identify relationships between the host and symbiont genetic distances and geography based on Spearman rank correlations. Pairwise genetic distances were calculated from both 18S rRNA gene sequences of the host and 16S rRNA gene sequences of the symbionts based on Kimura’s two-parameter model [81]. Geographic distances were determined by calculating the geodesics between sample sites with the GeoSphere package in R [82]. Host and symbiont sequences from *Trimyema* and *Caenomorpha* were excluded from this analysis because these ciliate hosts are phylogenetically divergent from the rest.

## Results

### Ultrastructure and localization of presumable methanogenic symbionts in ciliates

**Figure 1:**
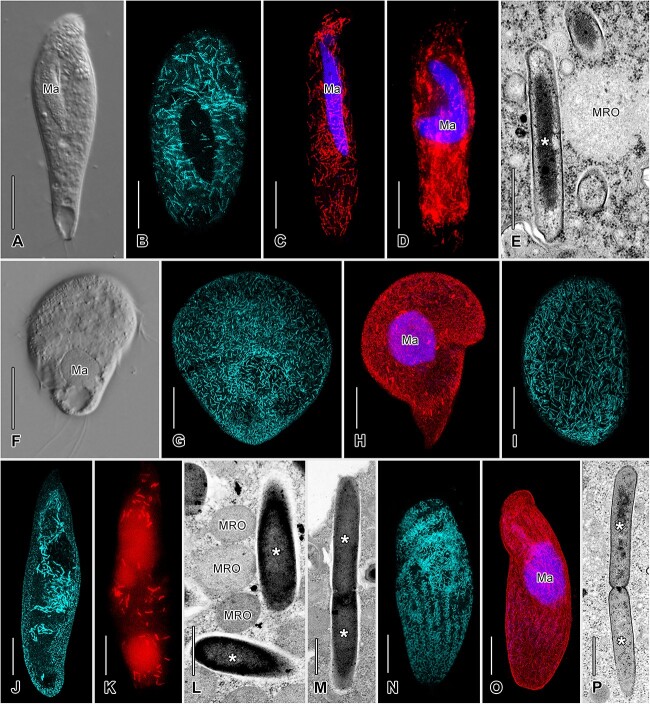
A–P, Representatives of studied anaerobic ciliates and their respective methanogenic endosymbionts. Ciliates shown *in vivo* (A, F), symbionts detected by FISH (C, D, H, K, O), micrographs showing methanogen-specific autofluorescence (B, G, I, J, N), and transmission electron micrographs showing the ultrastructure of the symbionts and host mitochondria (E, L, M, P). A–E, *Tropidoatractus levanderi* KAENG (B, C), RIOT (A, D), and KUCR13 (E) and its *Methanoregula* symbionts; methanogen-specific autofluorescence (B), cell hybridized with archaeal (C) and Methanomicrobiales-specific (D) probes, and ultrastructure of the symbionts in close proximity to host mitochondrion (E). F–H, *Urostomides bacillatus* (BOPAT) (F) and its *Methanobacterium* symbionts showing methanogen-specific autofluorescence (G) and hybridized with archaeal probe (H). I, Methanogenic symbionts of *Brachonella pulchra* (BOPAT) visualized by methanogen-specific autofluorescence. J–L, Methanogenic symbionts of *Bothrostoma undulans* (LERMA1); methanogen-specific autofluorescence (J), symbionts hybridized with archaeal probe (K), close association of the symbionts and host mitochondria (L), and dividing symbiont (M). N–P, Symbionts of *Metopus es*; symbionts of strain GDUKABAM showing methanogen-specific autofluorescence (N), hybridized with archaeal probe (O), and ultrastructure of strain TRIANGLE showing a diving symbiont (P). Macronucleus in FISH preparations was counterstained using DAPI. Ma, macronucleus; MOR, host's mitochondrion-related organelles; methanogenic symbionts marked by asterisks. Scale bars: E, L, M, P 500 nm; A–D, F–K, N, O 20 μm.

Methanogenic endosymbionts were detected by autofluorescence of the F420 coenzyme in 50 out of 54 of the studied strains (Figs. 1B, G, I, J, N, and S1; Table S10).

FISH using archaea- or Methanomicrobiales-specific oligonucleotide probes confirmed the presence of the methanogenic symbionts and further proved their distribution throughout the cytoplasm for selected strains of the host species *Tropidoatractus levanderi*, *Urostomides bacillatus*, *Bothrostoma undulans*, and *Metopus es* (Fig. 1C, D, H, K, O). Importantly, FISH experiments indicated the presence of the order Methanomicrobiales in the cytoplasm of *T. levanderi* since both the general archaeal probe ARC915 and MG1200b probe specific to order Methanomicrobiales showed a similar pattern (Fig. 1C, D), likely corresponding to its symbiont *Methanoregula* (Methanomicrobiales). Additionally, TEM revealed the ultrastructure and localization of the methanogenic symbionts in the cytoplasm of some strains belonging to the ciliates *T. levanderi*, *Bo*. *undulans*, and *M. es*. In all studied strains, the symbionts were found in close association with the ciliates’ MROs, and they were never surrounded by a host membrane (Fig. 1E, L, M, P). We observed thin rod-shaped symbiont cells with electron-dense centers in *T. levanderi* (KUCR13) (Fig. 1E) and wider, more electron-dense, symbiont cells in *Bo*. *undulans* (LERMA1) and *M. es* (TRIANGLE) (Fig. 1L, M, P). Dividing methanogens were also observed in *Bo*. *undulans* and *M. es* (Fig. 1M, P).

### A single methanogen dominates the archaeal community of each anaerobic ciliate

**Figure 2:**
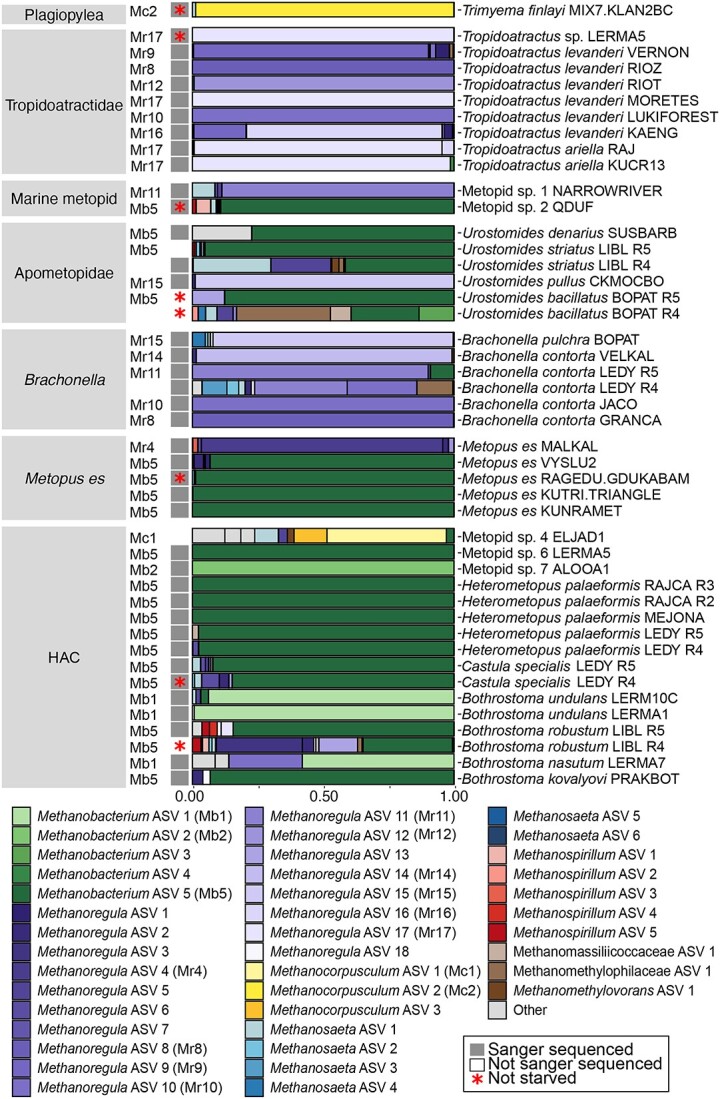
Barplot showing the relative abundances of the methanogen ASVs recovered from 16S rRNA gene amplicon sequencing from each ciliate sample. A dominant ASV is observed in most of the samples, which putatively represents the symbiotic methanogenic archaea. The dominant ASV (70% relative abundance) for each sample is indicated to the left of the bars, and these abbreviations are included in the legend as well. Mc: *Methanocorpusculum*, Mr: *Methanoregula*, and Mb: *Methanobacterium*. Non-methanogen ASVs are labeled “other.” Samples are grouped according to the host clade.

Methanogenic symbionts of all examined ciliate species, except *Palmarella salina*, were identified through Sanger (131 samples) and/or Illumina amplicon sequencing of the 16S rRNA gene (62 samples) (Supplementary methods). The Sanger sequencing resulted in clean, high-quality chromatograms, indicating the presence of a single dominant methanogenic archaeal genotype in each of the 131 samples, corresponding to 65 *Methanobacterium*, 56 *Methanoregula*, and 10 *Methanocorpusculum* sequences (Figs. S2, S3, and S4). All the Sanger sequences originated from the control medium were of poor quality, and with noisy background, e.g. ambiguous nucleotides and double peaks (not shown). The results from the amplicon Illumina sequencing of the ciliate samples were highly consistent with those from Sanger. Fifty-one amplicon samples were dominated by a single ASV corresponding to either *Methanoregula*, *Methanobacterium*, or *Methanocorpusculum,* each representing >70% of the amplicon reads, up to 100% in six samples (Figs. 2 and S5; Table S2). These dominant ASVs were identical to the Sanger sequence obtained from respective ciliate strains and consistent among replicates from the same strain (Table S7). Although the remaining amplicons recovered two or more ASVs, their dominant ASV, and in two cases the second dominant, was consistent with their respective Sanger sequences (Figs. 2 and S5; Table S2). Only one case, *Palmarella salina* WH5, the ASVs from the amplicon sequencing were incongruent with the Sanger sequencing results (Methanomethylophilaceae sp. vs. *Methanocorpusculum*, respectively, not shown).

**Figure 3:**
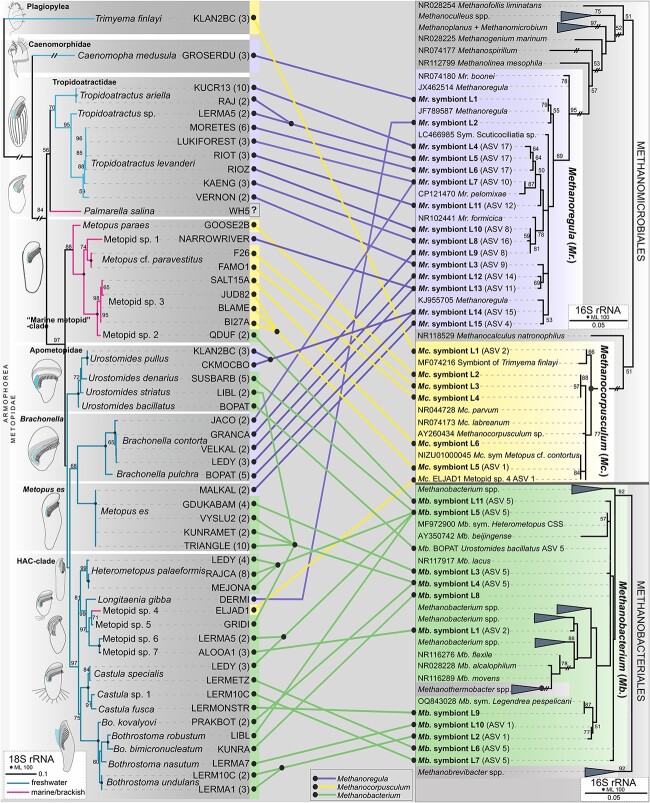
Maximum likelihood phylogenetic trees based on 18S (left) and 16S (right) rRNA gene sequences showing the connection between the 32 studied ciliate species and their respective methanogenic symbionts. The habitat of the host is depicted in the branches of the 18S tree. The number of 16S rRNA Sanger sequences obtained per ciliate strain is in brackets. The corresponding dominant ASVs which are 100% identical to the symbiont lineages are indicated in the 16S tree in brackets. Bootstrap values <50 are not shown. The scale bar represents 10 substitutions per 100 positions in the 18S tree and 5 substitutions per 100 positions in the 16S trees. For a full 18S tree, see the Supplemental Fig. S6. For full 16S trees, refer to the supplemental Figs. S2, S3, and S4.

From the 131 Sanger sequences, a total of 32 unique sequences were identified. These were considered lineages of *Methanoregula* (15), *Methanobacterium* (11), and *Methanocorpusculum* (6), corresponding to the 15 dominant ASVs recovered from amplicon sequencing—10 of *Methanoregula*, three of *Methanobacterium*, and two of *Methanocorpusculum* (Figs. 3, S2–4, S6, and S7). Based on the results generated by the Sanger and amplicon Illumina sequencing methods, we define those unique methanogen lineages (32) and their respective dominant ASVs (15) as putative symbionts for each strain and ciliate species (Figs. 3 and S6), except for *P. salina*, where the results were incongruent.

**Figure 4:**
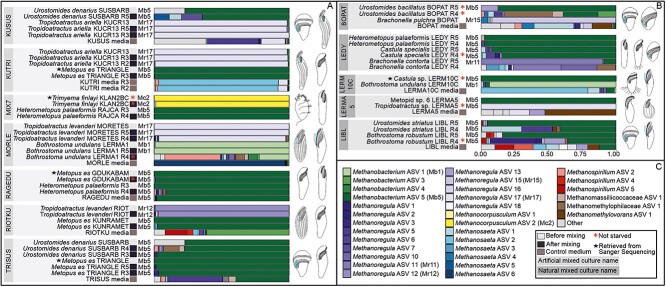
A, Barplot showing the relative abundances of methanogen ASVs recovered from 16S rRNA gene amplicon sequencing in the artificially mixed cultures, including the control medium. In each mixed culture, the combined ciliate species retained the same methanogen ASV before and after being mixed. Samples are grouped by mixed culture and boxes to the left of the barplots indicate whether the time the sample was taken (before or after mixing). B, Relative abundances of methanogen ASVs in natural mixed cultures which contained naturally co-occurring ciliate species. Samples are grouped by the culture name. Stars indicate that there was no amplicon sequencing performed on the sample, but that the results from Sanger sequencing (the ASV which is identical to the 16S rRNA gene sequence obtained from Sanger) are represented in the barplot instead. Asterisks indicate that host cells were not starved during sample processing.

### Symbiont specificity within a host species

Based on the sequencing results, all the identified methanogenic symbionts from the replicate samples that originated from one ciliate strain were genetically identical (Figs. S2, S3, S4, and S8A; Table S12). The methanogens from different strains of a particular ciliate species, except for *M. es*, always belonged to the same methanogen genus, but their 16S rRNA Sanger sequences usually differed (Figs. 3, S6, and S8B, D). The methanogen lineages were 100% identical across all studied strains only in *Bo*. *undulans*, *Heterometopus palaeformis*, and *U. pullus* (Figs. 3 and S6). Eight of the identified symbiont lineages (four *Methanoregula*, one *Methanocorpusculum*, and three *Methanobacterium*) were shared between different strains, species, or genera of anaerobic ciliates. Remarkably, the remaining 24 symbiont lineages were exclusively found in a single ciliate strain (Figs. 3 and S6).

### Methanogen genera across the ciliate host phylogeny and environment

We studied anaerobic ciliates from two classes—Armophorea, with 31 species, in two orders (Armophorida and Metopida), and Plagiopylea, represented by a single species (*Trimyema finlayi*). The 30 Metopida species form six clades, here referred to as Tropidoatractidae, “marine metopid clade,” Apometopidae, *Brachonella* clade, *M. es* clade, and HAC clade (Figs. 3 and S6). The Armophorida are represented by *Caenomorpha medusula*.

The distribution of methanogenic symbionts across phylogeny (clade/genus) and habitat (marine/brackish vs. freshwater) of the host showed a degree of specificity (Figs. 3, S6, and S8D). *Methanobacterium* and *Methanoregula* symbionts were detected in all the freshwater armophorean species (metopids and *C*. *medusula*), in the single soil metopid (ALOOA1), and in two marine/brackish species from the “marine metopid clade” (NARROWRIVER and QDUF). In contrast, *Methanocorpusculum* was found in the rest of the marine/brackish metopid species, except in *P. salina* (Tropidoatractidae), and in the freshwater plagiopylid *Trimyema finlayi*.

In most cases, a particular methanogen genus was consistently observed across all species of a particular ciliate genus or clade (Fig. 3). For instance, *Methanoregula* was present in all the examined species and strains of *Tropidoatractus* (Tropidoatractidae), *Brachonella* (*Brachonella* clade), *Longitaenia* (HAC clade), and *Caenomorpha* (Caenomorphidae), whereas *Methanobacterium* was observed in almost all the strains within the HAC clade, e.g. *Heterometopus*, *Castula*, and *Bothrostoma*. The exceptions were the genus *Urostomides* (Apometopidae), where the examined species possessed different methanogens (*Methanobacterium* in *U*. *denarius* and *U. striatus*, and *Methanoregula* in *U. pullus*) and the species *M. es*, in which one strain (MALKAL) harbored *Methanoregula*, while the remaining four harbored *Methanobacterium* (Figs. 3, S6, S7, and S8D).

The degree of specificity that we observed between host and symbiont was further supported statistically—full and partial Mantel tests indicated a weak, but significant, positive correlation between host and symbiont genetic distances (*r* = 0.1529, *P* = 0.001), whereas there was no observed association between symbiont genetic distances and geography (Table S13). This suggests that host specificity is more important for symbiont variation than geography.

### Symbiont switches do not occur in experimentally co-cultured ciliates

A total of seven artificially mixed cultures, each containing a pair of different ciliate species, were successfully established. Five of them contained species that harbored different methanogenic symbionts (*Methanobacterium* vs. *Methanoregula*) before mixing (Table S1, Figs. 4 and S8C). During our experiments, the sequencing results revealed that the dominant methanogenic symbiont ASV associated with each ciliate taxon remained the same, even when grown in the presence of another ciliate and its associated symbionts (Fig. 4A). Due to sparse read numbers obtained during amplicon sequencing of *Trimyema finlayi* KLAN2BC and *M. es* TRIANGLE prior to mixing, we instead compared the Sanger sequences obtained from both species with the dominant ASV obtained after they were mixed, resulting in 100% identity between Sanger sequences and ASVs. Despite our observation that *M. es* harbors either *Methanobacterium* or *Methanoregula*, after mixing a *Methanobacterium*-hosting strain of *M. es* with *Methanoregula*-specific ciliate species (e.g. *Tropidoatractus* spp.), no switches of methanogens and ASVs were observed in either partner.

Except for the MIX7 culture, the free-living archaeal community from the control medium of these mixed cultures (Fig. 4A) as well as for four naturally mixed cultures (BOPAT, LERMA5, LERM10C, LIBL; Fig. 4B) was also screened to determine the methanogen community persisting outside the ciliates (Fig. 4). For the RAGEDU culture, a single dominant ASV (*Methanobacterium* sp. ASV5) was observed in both the control medium and the two mixed ciliates. In the remaining cultures, the archaeal communities and their relative abundance of the ciliates differed from those in the control medium, e.g. the ASVs belonging to the genus *Methanosaeta* were dominant in KUTRI and MORLE. The presence of the symbiont ASV in the medium, i.e. in RAGEDU culture, might be possibly attributed to its persistence or its accidental presence after being released from dead host cells.

## Discussion

### Anaerobic ciliates maintain stable populations of dominant methanogenic symbionts

Based on our and previous observations [11, 45], metopid cells typically host hundreds to thousands of symbiotic methanogens (Table S10). We observed the symbionts living adjacent to the host’s MROs (Fig. 1E, L) [27, 35, 36], which supports the hypothesis that they are metabolically integrated through hydrogen-based syntrophy [6, 7, 54]. Previously, the symbionts of only a limited number of ciliate populations were identified mostly via Sanger sequencing [12, 28–32, 35, 37, 38]. Here, we found that each ciliate strain (except for *Palmarella salina* WH5) hosts an archaeal community, which is dominated (>70% relative abundance) by a single methanogen ASV, and this methanogen ASV is identical to the 16S rRNA gene sequence obtained via Sanger sequencing from the same ciliate strain. These results strongly corroborate previous suggestions that a particular anaerobic ciliate population is generally colonized by a single, dominant methanogen genotype [12, 28, 29].

Most of the symbiotic methanogens of free-living ciliates and other anaerobic protists belong to *Methanobacterium*, *Methanoregula*, and *Methanocorpusculum* [6, 12, 18, 28–30, 35, 37, 38, 44]. Besides being intracellular symbionts of anaerobic protists, members of these genera are also found as luminal symbionts in the guts of various invertebrates and vertebrates, as well as free-living in a wide range of anoxic environments [22, 33, 83–88]. These genera belong to different euryarchaeote classes, Methanobacteria (*Methanobacterium*) and Methanomicrobia (*Methanoregula* and *Methanocorpusculum*), but share a hydrogenotrophic metabolism, i.e. they all are dependent on H_2_ intake and, therefore, are likely to play a syntrophic role dependent on the use of H_2_ from ciliate fermentation [9, 13, 18, 19, 22, 36, 38, 88–90].

Interestingly, the methanogenic symbionts identified here and those from other studies [18, 27, 30, 32, 36] are different from their free-living relatives. However, they cluster among them in a non-monophyletic manner (Figs. 3 and S7), implying that methanogen acquisition from the environment by anaerobic protists is possible, as suggested previously [11, 12] and experimentally demonstrated with the successful acquisition and integration of a *Methanobacterium* into an aposymbiotic strain of the ciliate *Trimyema compressum* through ingestion without digestion [57].

### The host lifestyle and habitat are crucial drivers of the ciliate–methanogen specificity

Although evidence of co-diversification between anaerobic ciliates and their methanogenic endosymbionts has not been observed [6, 12, 30] (Fig. 3), our extensive taxon sampling has identified host lifestyle and habitat as factors which appear to influence the specificity of these partnerships. The methanogens from endobiotic ciliates are different from those of the free-living ciliates [12, 22, 91]. Two unrelated lineages of endobiotic ciliates, Trichostomatia (rumen ciliates) and Clevelandellida (e.g. *Nyctotherus*), harbor *Methanobrevibacter* (Methanobacteriales) [12, 91, 92]. *Methanobrevibacter* is also a predominant methanogen found in the gut, intestines, and rumen [91, 93]. As a symbiont of free-living ciliates, it has been only found in *Trimyema compressum* (Plagiopylea) [43]. Interestingly, the free-living close relatives of both, Clevelandellida (i.e. metopids) and Trichostomatia (i.e. *Legendrea* and *Dactylochlamys* [37]), invariantly host *Methanoregula*, *Methanocorpusculum*, *Methanosaeta*, or *Methanobacterium*. This suggests that when an anaerobic ciliate becomes endobiotic, a symbiont switch takes place, in this case to *Methanobrevibacter* (Fig. S8D).

We also observed specificity patterns in methanogenic symbionts of free-living metopids isolated from two contrasting environments. We found that freshwater metopids are specific to *Methanobacterium* or *Methanoregula*. Based on environmental sequencing, *Methanoregula* and *Methanobacterium* are typically freshwater and rare in marine environments [84, 85, 87, 94]. This underscores the presence of these methanogens as symbionts of anaerobic protists in freshwater ecosystems [12, 18, 27, 30, 37]. In contrast, most of the marine strains from the “marine metopid clade,” including the previously studied *Metopus contortus* [28, 36, 44] and the only marine metopid strain (ELJAD1) from the predominantly freshwater HAC clade, specifically host *Methanocorpusculum* (Figs. 3, S4, S6, S7, and S8D). Moreover, *Methanocorpusculum*, absent in the freshwater metopids, apparently dominates as a symbiont of marine/brackish metopids and plagiopylids [44]. Nevertheless, exceptions exist—three metopids in the “marine metopid clade,” two of these originating from marine/brackish environments and one from granular sludge [32], host *Methanobacterium* or *Methanoregula* (Fig. 3). Despite the observed exceptions, and the potential absence of methanogens in *P. salina*, our data strongly support the idea that the environment is involved in the ciliate–methanogen specificity.

Other available yet scarce data suggest that the role of habitat (i.e. freshwater or marine/brackish) in metopid’s specificity for a particular methanogen may not apply to other lineages of anaerobic ciliates [30–32, 35, 37, 43]. For instance, *Methanocorpusculum* was found as a symbiont of two unrelated freshwater ciliate species: *Trimyema finlayi* (Plagiopylea) [31, 35] and *Dactylochlamys pisciformis* (Litostomatea) [37], but their closest relatives, which are also freshwater species, host a different methanogen, i.e. *Methanobrevibacter* in *Trimyema compressum* [43], and *Methanobacterium* or *Methanosaeta* in *Legendrea* spp. [37].

### The taxonomy of the host also influences the methanogen specificity

So far, co-diversification has not been observed between anaerobic protists and their methanogenic symbionts, suggesting a lack of strict vertical transmission [6, 11, 12, 30, 32]. However, we have found via a broad taxon sampling that in metopids there is considerable partner specificity [95] at the broader host taxonomy level. Although the phylogeny of metopids has not yet been completely resolved [96, 97], 18S rRNA gene phylogenies consistently recover the main metopid groups [96] (Fig. S6). Here, we divided the metopid species into six robust clades and assessed the identity of the methanogenic symbionts of multiple isolates within each clade. When the strong effect of the environment (i.e. freshwater vs. marine/brackish metopids) was filtered out and only freshwater metopids were considered, an interesting pattern suggesting specificity at the host clade level became apparent. Five of the six clades seem to be highly specific to a particular methanogen genus: all freshwater strains of Tropidoatractidae and *Brachonella* hosted *Methanoregula*, whereas *M. es* and the HAC clade show clear preference for *Methanobacterium* (Figs. S7 and S8D).

The methanogen specificity at a higher taxon level might also be corroborated in future studies by determining the identity of symbionts of so-far unstudied metopid genera (e.g. *Planometopus* and *Atopospira*). Interestingly, the studied metopids from the mixed cultures, as well as those from naturally co-occurring cultures (Table S1), demonstrated fidelity, and, therefore, specificity at the clade level, for certain methanogen genera since both partners retained their methanogens during the experiment (Fig. 4). The situation in *Urostomides* (Apometopidae), and likely *Castula* (HAC), is less clear and we cannot discard the possibility that these genera are more opportunistic, certainly being good candidates for further studies (Figs. S6, S7, and S8D).

### Lack of methanogen specificity at the ciliate species level

Despite a strong fidelity [95] between host genera/clade, the situation differs considerably at the host species level. The behavior of symbioses between ciliates and prokaryotes varies from almost strict co-diversification, as seen in the ciliate *Kentrophoros* and its thiotrophic symbionts [56], to frequent symbiont replacement, such as in *Euplotes* ciliates and their multiple symbionts [98–100]. Our results show that within a ciliate species, the methanogen symbionts are closely related but genetically divergent (Fig. 3). Similar results have been reported in metopids and plagiopylids [44]. However, a few promiscuous methanogen lineages, which associated with a large diversity of ciliate hosts, were also observed in ciliate species belonging to the same genus (*Tropidoatractus*) or even from different genera (marine metopids, *Tropidoatractus*, *Brachonella*, *Urostomides*). Notably, ciliate hosts that shared a particular methanogen lineage came from different sampling sites, with some originating even from different continents. Inversely, different metopid species retrieved from the same sampling site and at the same time harbored distinct methanogen lineages (https://shorturl.at/IUuqI), e.g. ciliates from Atarasquillo, Mexico. Similar findings were already documented [12] though in a considerably lower number of taxa. This implies that ciliates likely do not acquire the most abundant methanogen from the environment and suggests some level of symbiont–host fidelity and specificity. This is supported by the Mantel test results (Table S13), where we found no significant correlation between symbiont genetic distances and the geographic distances between samples. We did find that host and symbiont genetic distances are positively correlated (full test *r* = 0.1689, *P* = 0.001; partial test controlling for geography *r* = 0.1529, *P* = 0.001). Taken together, these results suggest that host specificity is more important for symbiont variation than geography and supports our hypothesis of host–symbiont specificity at some, but not all, host taxonomic levels.

Three of our retrieved symbiont lineages were identical to already published symbiont sequences from two metopids (*Heterometopus* sp. CSS, *Metopus* cf. *contortus*) and one litostomatean (*Legendrea pespelicani*) [27, 36, 37]. Furthermore, one of the *Methanoregula* lineages found in *T. levanderi* and *B. contorta* was nearly identical (two nucleotide differences) to the endosymbiotic *Ca. Methanoregula pelomyxae* isolated from the archamoeba *Pelomyxa schiedti* [18] (Figs. 3 and S6). This alludes to the fact that there may be promiscuous methanogen lineages which can be acquired by distantly related anaerobic protists.

In the case of *H. palaeformis* and *U. pullus*, we observed a strict specificity pattern since all the strains of each species harbored a unique methanogen lineage. Interestingly, the symbiont lineage of herein studied *H. palaeformis* is identical to the methanogen symbiont of *Heterometopus* sp. CSS [27], but differs from the symbiont previously detected in a different population of *H*. *palaeformis* [29]. On the other hand, the methanogen lineage found in the two examined strains of *U. pullus* was also found in *Brachonella pulchra*, hinting a lack of specificity at the intraspecies host level in metopid ciliates.

Strong specificity was also found in the case of *Trimyema finlayi* and its *Methanocorpusculum* symbiont since the obtained Sanger sequence from this study (Czech Republic) is highly similar (99.45% identity) to the one from the UK [31, 35]. Phylogenetically, the sequences of *Methanocorpusculum* symbionts are distributed in two clades: one of them comprises solely the symbionts of *Trimyema finlayi*, whereas the second clade clusters all the symbionts from marine/brackish metopids, including one of *Metopus* cf. *contortus* [36], together with some free-living methanogens (Figs. 3 and S4). Nevertheless, it was shown recently that the *Methanocorpusculum* symbionts of marine relatives of *Trimyema finlayi*, and *Plagiopyla*, are distributed in a non-monophyletic pattern and cluster within those of the marine metopids, suggesting a weak specificity [44].

Altogether, our data suggest that strict specificity patterns and co-diversification are uncommon between anaerobic ciliates and methanogens, fitting more into a symbiont replacement scheme in which methanogens are potentially replaced through horizontal transmission, like in the case of *Euplotes* [100].

### The methanogen–ciliate association implies a mixed transmission mode

At short evolutionary timescales, vertical transmission during host division is a straightforward mechanism to maintain symbiont fidelity across generations, as previously demonstrated in the ciliate *Plagiopyla frontata* [55]. Moreover, we also observed dividing methanogenic symbionts within the host cytoplasm (Fig. 1M, P). This hypothesis is supported by the fact that symbiont sequences are identical across samples originating from a particular ciliate strain (Fig. S8A). Additionally, our co-cultivation experiments indicate that the ciliate–methanogen relationship is stable over short timescales since symbiont switches between host partners were not detected, and the methanogens from the environment apparently do not easily associate with the host, perhaps due to priority effect. This implies that the fidelity of a ciliate strain to its methanogenic symbiont is very strong.

Even so, occasional horizontal transmission of methanogens apparently also occurs among closely related hosts (e.g. within a species). Remarkably, the different methanogen lineages observed in each strain of a particular ciliate species, except for *M. es*, were closely related and always belonged to the same methanogen genus displaying not only the specificity of a ciliate species to a typically one methanogen genus, but also the capacity for flexibility and symbiont exchange or reacquisition through horizontal transmission as observed in the *Euplotes* symbiosis system [100]. Horizontal transmission would facilitate the substitution of a current symbiont lineage with a more optimal strain when environmental or other conditions change, the replacement of symbionts that have degenerated through genome erosion, and/or the reacquisition of symbionts upon loss of their partner [57, 58, 98–100]. Altogether, this implies a “mixed-mode” transmission system, where vertical transmission is common over short timescales, but horizontal transmission occurs occasionally [95, 101, 102].

### Further considerations

Our extensive survey of anaerobic ciliates and their methanogens, focused on the order Metopida and including most of its lineages, greatly expands our knowledge of this ecologically important symbiosis, elucidating specificity patterns across the host’s phylogeny, environment, and lifestyle. Mechanisms involved in the establishment and maintenance of a dominant methanogen within a host ciliate have not been revealed but might include small methanogen population sizes during the initial stages of infection, symbiont population bottleneck during vertical transmission, recognition-based control of the infection by the host or symbiont leading to eradication of nonspecific symbionts, or incompatibility of nonspecific methanogens with the host’s intracellular environment. Also, intra-host competition and priority effects should be considered since the volume of the host’s cytoplasm is limited, as is the space around the MROs available for the methanogens. Already established symbionts may thus occupy all convenient spots and prevent colonization by other methanogen lineages.

Although the transmission modes of the methanogens are not yet fully understood, it is likely that a ciliate population maintains its symbiotic methanogen lineage through vertical transmission during the host cell division, which results in a strong partner fidelity. Additionally, the symbionts within a host species are genetically divergent without co-diversification, possibly due to occasional replacements through horizontal transmission, but showing a strong partner specificity.

The identified methanogenic symbiont lineages, along with previously sequenced symbionts, were genetically different yet similar to the “putatively” free-living methanogens, but clustered together without distinction on their lifestyle (Fig. 3). Regardless, the resolution of our phylogeny of the methanogens is weak due to a short, sequenced fragment of the 16S rRNA gene, which could obscure co-diversification patterns, if any exist. Phylogenomics may be necessary to fully resolve the relationships among closely related methanogenic symbionts of anaerobic ciliates. The acquisition/replacement of the methanogenic symbiont by a ciliate potentially may occur during an uptake from the environment, direct ingestion, or through eukaryovory. Conjugation is an alternative process in which ciliate partners may theoretically exchange their methanogenic symbionts, although evidence is unavailable. If it occurs, then it could be strong evidence of horizontal transmission, and, furthermore, evidence for a strong fidelity and specificity for a particular methanogen genus. Ciliates devoid of methanogens, naturally or experimentally, should also be studied using molecular tools to determine potential symbiont acquisitions through ingestion. The mechanisms involved in the symbiont replacement are unknown in anaerobic ciliates; however, the environment generally appears to play a large role since our results suggest that ciliates possibly switch their methanogens with those that are present in their niche, i.e. endobiotic, freshwater, or marine. Future studies focusing on monitoring ciliate–methanogen symbioses in natural populations through time could further unveil the level of host specificity.

## Supplementary Material

Supplementary_material

## Data Availability

Raw 16S rRNA gene amplicon sequences are available in the Sequence Read Archive (SRA) on NCBI (BioProject ID PRJNA1071061). The 18S rRNA gene sequences of the studied ciliates as well as their 16S rRNA from their methanogenic symbionts are deposited in GenBank under the accession numbers PP274949–PP274988, PP935522–PP935534, and PP250167–PP250287, respectively. Unaligned, aligned, and trimmed datasets used for the analyses are in the Supplementary files.
