# Supplementary material for: Methanogenic symbionts of anaerobic ciliates are host and habitat specific
Source: ISME J. 2024 Aug 20;18(1):wrae164. doi: 10.1093/ismejo/wrae164 (PMC11378729; doi:10.1093/ismejo/wrae164)
Supplement: Supplementary_material [file supplementary_material.zip › FigureS1_Autofluorescence.pdf]

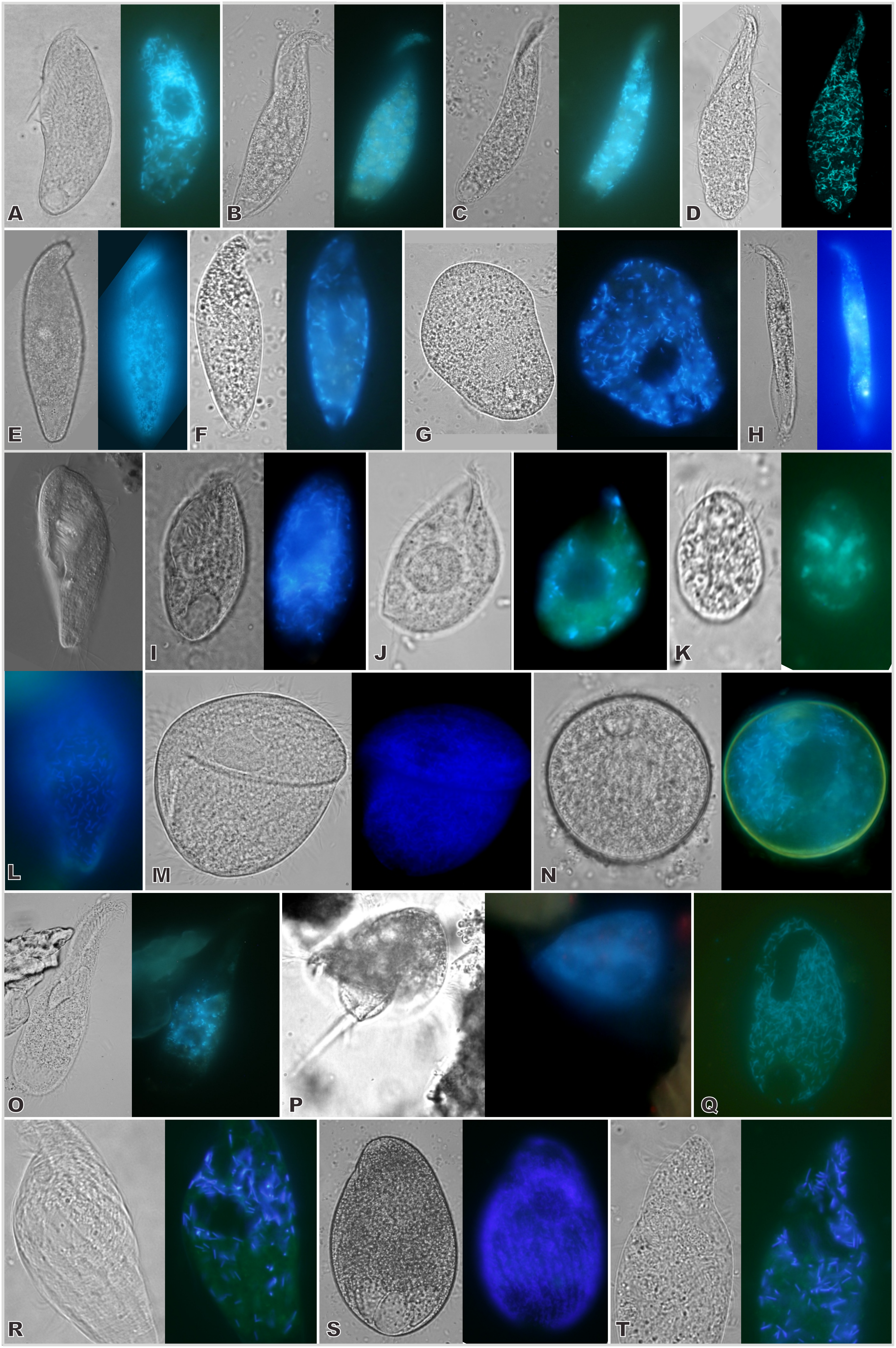
A
B
C
D
E
F
G
H
I
J
K
L
M
N
O
P
Q
R
S
T

Figure S1. Anaerobic ciliates in bright field (left or up) and under autofluorescence (right or down) showing the rod-shaped methanogenic symbionts. D. Confocal microscopy. A, *Bothrosrtoma undulans* LERMA1. B, Metopid sp. 7 ALOOA1. C–D, *Heterometopus palaeformis* RAJCA before (C) and after mixing (D). E, *Metopus es* GDUKABAM. F, *Tropidoatractus ariella* KUCR13. G, *Urostomides denarius* SUSBARB. H, Metopid sp. 4 ELJAD1. I, *Bothrostoma nasutum* LERMA7. J, *Tropidoatractus* sp. LERMA5. K, *Trimyema finlayi* KLAN2BC. L, *Bothrostoma robustum* LIBL. M, *Brachonella pulchra* BOPAT. N, Cyst of *Urostomides bacillatus* BOPAT. O, Metopid sp. 5 LERMA5. P, *Caenomorpha medusula* GROSERDU. Q, *Tropidoatractus levanderi* VERNON. R, *Tropidoatractus ariella* RAJ. S, *Metopus es* TRIANGLE. T, *Heterometopus palaeformis* MEJONA.
