# Supplementary material for: Methanogenic symbionts of anaerobic ciliates are host and habitat specific
Source: ISME J. 2024 Aug 20;18(1):wrae164. doi: 10.1093/ismejo/wrae164 (PMC11378729; doi:10.1093/ismejo/wrae164)
Supplement: Supplementary_material [file supplementary_material.zip › FigureS2_MethanobacteriumTree.pdf]

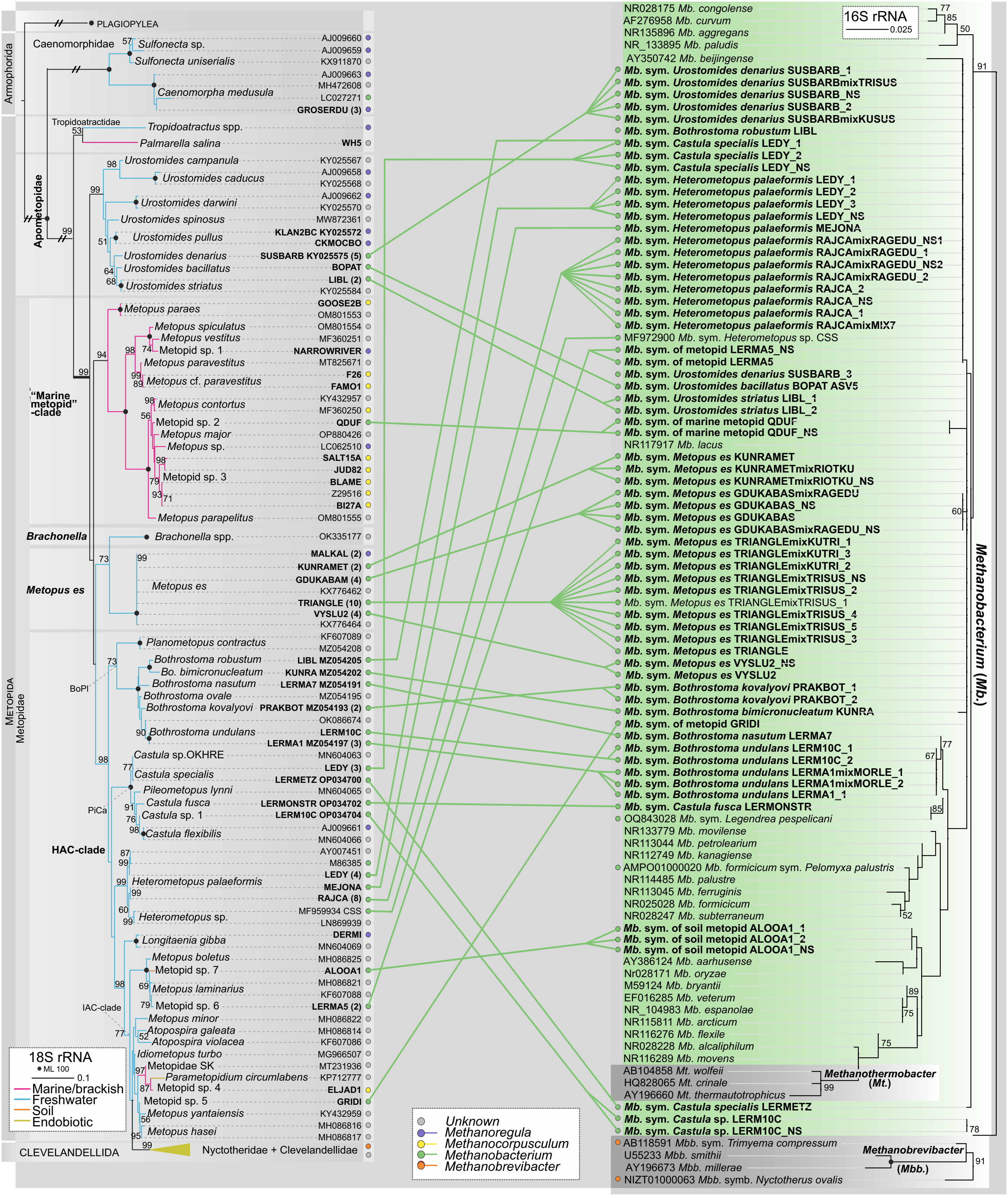

PLAGIOPYLEA
Armophorida
Caenomorphidae
Sulfonecta sp.
Sulfonecta uniserialis
Caenomorpha medusula
AJ009660
AJ009659
KX911870
AJ009663
MH472608
LC027271
GROSERDU (3)
Tropidoatractidae
Tropidoatractus spp.
Palmarella salina
WH5
Apometopidae
Urostomides campanula
Urostomides caducus
Urostomides darwini
Urostomides spinosus
Urostomides pullus
Urostomides denarius
Urostomides bacillatus
Urostomides striatus
KY025567
AJ009658
KY025568
AJ009662
KY025570
MW872361
KLAN2BC KY025572
CKMOCBO
SUSBARB KY025575 (5)
BOPAT
LIBL (2)
KY025584
"Marine metopid" -clade
Metopus paraes
Metopus spiculatus
Metopus vestitus
Metopid sp. 1
Metopus paravestitus
Metopus cf. paravestitus
Metopus contortus
Metopid sp. 2
Metopus major
Metopus sp.
Metopid sp. 3
Metopus parapelitus
GOOSE2B
OM801553
OM801554
MF360251
NARROWRIVER
MT825671
F26
FAMO1
KY432957
MF360250
QDUF
OP880426
LC062510
SALT15A
JUD82
BLAME
Z29516
BI27A
OM801555
Brachonella
Brachonella spp.
OK335177
Metopus es
MALKAL (2)
KUNRAMET (2)
GDUKABAM (4)
KX776462
TRIANGLE (10)
VYSLU2 (4)
KX776464
METOPIDA
Metopidae
BoPl
Planometopus contractus
Bothrostoma robustum
Bo. bimicronucleatum
Bothrostoma nasutum
Bothrostoma ovale
Bothrostoma kovalyovi
Bothrostoma undulans
KF607089
MZ054208
LIBL MZ054205
KUNRA MZ054202
LERMA7 MZ054191
MZ054195
PRAKBOT MZ054193 (2)
OK086674
LERM10C
LERMA1 MZ054197 (3)
PiCa
Castula sp.OKHRE
Castula specialis
Pileometopus lynni
Castula fusca
Castula sp. 1
Castula flexibilis
MN604063
LEDY (3)
LERMETZ OP034700
MN604065
LERMONSTR OP034702
LERM10C OP034704
AJ009661
MN604066
HAC-clade
Heterometopus palaeformis
Heterometopus sp.
AY007451
M86385
LEDY (4)
MEJONA
RAJCA (8)
MF959934 CSS
LN869939
Longitaenia gibba
DERMI
MN604069
IAC-clade
Metopus boletus
Metopid sp. 7
Metopus laminarius
Metopid sp. 6
Metopus minor
Atopospira galeata
Atopospira violacea
Idiometopus turbo
Metopidae SK
Parametopidium circumlabens
Metopid sp. 4
Metopid sp. 5
Metopus yantaiensis
Metopus hasei
MH086825
ALOOA1
MH086821
KF607088
LERMA5 (2)
MH086822
MH086814
KF607086
MG966507
MT231936
KP712777
ELJAD1
GRIDI
KY432959
MH086816
MH086817
CLEVELANDELLIDA
Nyctotheridae + Clevelandellidae
18S rRNA
ML 100
0.1
Marine/brackish
Freshwater
Soil
Endobiotic
Unknown
Methanoregula
Methanocorpusculum
Methanobacterium
Methanobrevibacter
16S rRNA
0.025
NR028175 Mb. congolense
AF276958 Mb. curvum
NR135896 Mb. aggregans
NR_133895 Mb. paludis
AY350742 Mb. beijingense
Mb. sym. Urostomides denarius SUSBARB_1
Mb. sym. Urostomides denarius SUSBARBmixTRISUS
Mb. sym. Urostomides denarius SUSBARB_NS
Mb. sym. Urostomides denarius SUSBARB_2
Mb. sym. Urostomides denarius SUSBARBmixKUSUS
Mb. sym. Bothrostoma robustum LIBL
Mb. sym. Castula specialis LEDY_1
Mb. sym. Castula specialis LEDY_2
Mb. sym. Castula specialis LEDY_NS
Mb. sym. Heterometopus palaeformis LEDY_1
Mb. sym. Heterometopus palaeformis LEDY_2
Mb. sym. Heterometopus palaeformis LEDY_3
Mb. sym. Heterometopus palaeformis LEDY_NS
Mb. sym. Heterometopus palaeformis MEJONA
Mb. sym. Heterometopus palaeformis RAJCAmixRAGEDU_NS1
Mb. sym. Heterometopus palaeformis RAJCAmixRAGEDU_1
Mb. sym. Heterometopus palaeformis RAJCAmixRAGEDU_NS2
Mb. sym. Heterometopus palaeformis RAJCAmixRAGEDU_2
Mb. sym. Heterometopus palaeformis RAJCA_2
Mb. sym. Heterometopus palaeformis RAJCA_NS
Mb. sym. Heterometopus palaeformis RAJCA_1
Mb. sym. Heterometopus palaeformis RAJCAmixMIX7
MF972900 Mb. sym. Heterometopus sp. CSS
Mb. sym. of metopid LERMA5_NS
Mb. sym. of metopid LERMA5
Mb. sym. Urostomides denarius SUSBARB_3
Mb. sym. Urostomides bacillatus BOPAT ASV5
Mb. sym. Urostomides striatus LIBL_1
Mb. sym. Urostomides striatus LIBL_2
Mb. sym. of marine metopid QDUF
Mb. sym. of marine metopid QDUF_NS
NR117917 Mb. lacus
Mb. sym. Metopus es KUNRAMET
Mb. sym. Metopus es KUNRAMETmixRIOTKU
Mb. sym. Metopus es KUNRAMETmixRIOTKU_NS
Mb. sym. Metopus es GDUKABASmixRAGEDU
Mb. sym. Metopus es GDUKABAS_NS
Mb. sym. Metopus es GDUKABAS
Mb. sym. Metopus es GDUKABASmixRAGEDU_NS
Mb. sym. Metopus es TRIANGLEmixKUTRI_1
Mb. sym. Metopus es TRIANGLEmixKUTRI_3
Mb. sym. Metopus es TRIANGLEmixKUTRI_2
Mb. sym. Metopus es TRIANGLEmixTRISUS_NS
Mb. sym. Metopus es TRIANGLEmixTRISUS_2
Mb. sym. Metopus es TRIANGLEmixTRISUS_1
Mb. sym. Metopus es TRIANGLEmixTRISUS_4
Mb. sym. Metopus es TRIANGLEmixTRISUS_5
Mb. sym. Metopus es TRIANGLEmixTRISUS_3
Mb. sym. Metopus es TRIANGLE
Mb. sym. Metopus es VYSLU2_NS
Mb. sym. Metopus es VYSLU2
Mb. sym. Bothrostoma kovalyovi PRAKBOT_1
Mb. sym. Bothrostoma kovalyovi PRAKBOT_2
Mb. sym. Bothrostoma bimicronucleatum KUNRA
Mb. sym. of metopid GRIDI
Mb. sym. Bothrostoma nasutum LERMA7
Mb. sym. Bothrostoma undulans LERM10C_1
Mb. sym. Bothrostoma undulans LERM10C_2
Mb. sym. Bothrostoma undulans LERMA1mixMORLE_1
Mb. sym. Bothrostoma undulans LERMA1mixMORLE_2
Mb. sym. Bothrostoma undulans LERMA1_1
Mb. sym. Castula fusca LERMONSTR
OQ843028 Mb. sym. Legendrea pespelicani
NR133779 Mb. movilense
NR113044 Mb. petrolearium
NR112749 Mb. kanagiense
AMPO01000020 Mb. formicicum sym. Pelomyxa palustris
NR114485 Mb. palustre
NR113045 Mb. ferruginis
NR025028 Mb. formicicum
NR028247 Mb. subterraneum
Mb. sym. of soil metopid ALOOA1_1
Mb. sym. of soil metopid ALOOA1_2
Mb. sym. of soil metopid ALOOA1_NS
AY386124 Mb. aarhusense
Nr028171 Mb. oryzae
M59124 Mb. bryantii
EF016285 Mb. veterum
NR_104983 Mb. espanolae
NR115811 Mb. arcticum
NR116276 Mb. flexile
NR028228 Mb. alcaliphilum
NR116289 Mb. movens
AB104858 Mt. wolfeii
HQ828065 Mt. crinale
AY196660 Mt. thermautotrophicus
Methanothermobacter (Mt.)
Mb. sym. Castula specialis LERMETZ
Mb. sym. Castula sp. LERM10C
Mb. sym. Castula sp. LERM10C_NS
Methanobacterium (Mb.)
AB118591 Mbb. sym. Trimyema compressum
U55233 Mbb. smithii
AY196673 Mbb. millerae
NIZT01000063 Mbb. symb. Nyctotherus ovalis
Methanobrevibacter (Mbb.)

Figure S2. Maximum likelihood phylogenetic trees based on 18S (left) and 16S (right) rRNA gene sequences showing the connection between ciliate hosts and all obtained symbiotic Sanger sequences belonging to the genus *Methanobacterium* (in bold). The habitat of the ciliate is depicted in the 18S tree. The scale bar represents 5 substitutions per 100 positions. Bootstrap values below 50 are not shown.
