# Supplementary material for: Methanogenic symbionts of anaerobic ciliates are host and habitat specific
Source: ISME J. 2024 Aug 20;18(1):wrae164. doi: 10.1093/ismejo/wrae164 (PMC11378729; doi:10.1093/ismejo/wrae164)
Supplement: Supplementary_material [file supplementary_material.zip › FigureS3_MethanoregulaTree.pdf]

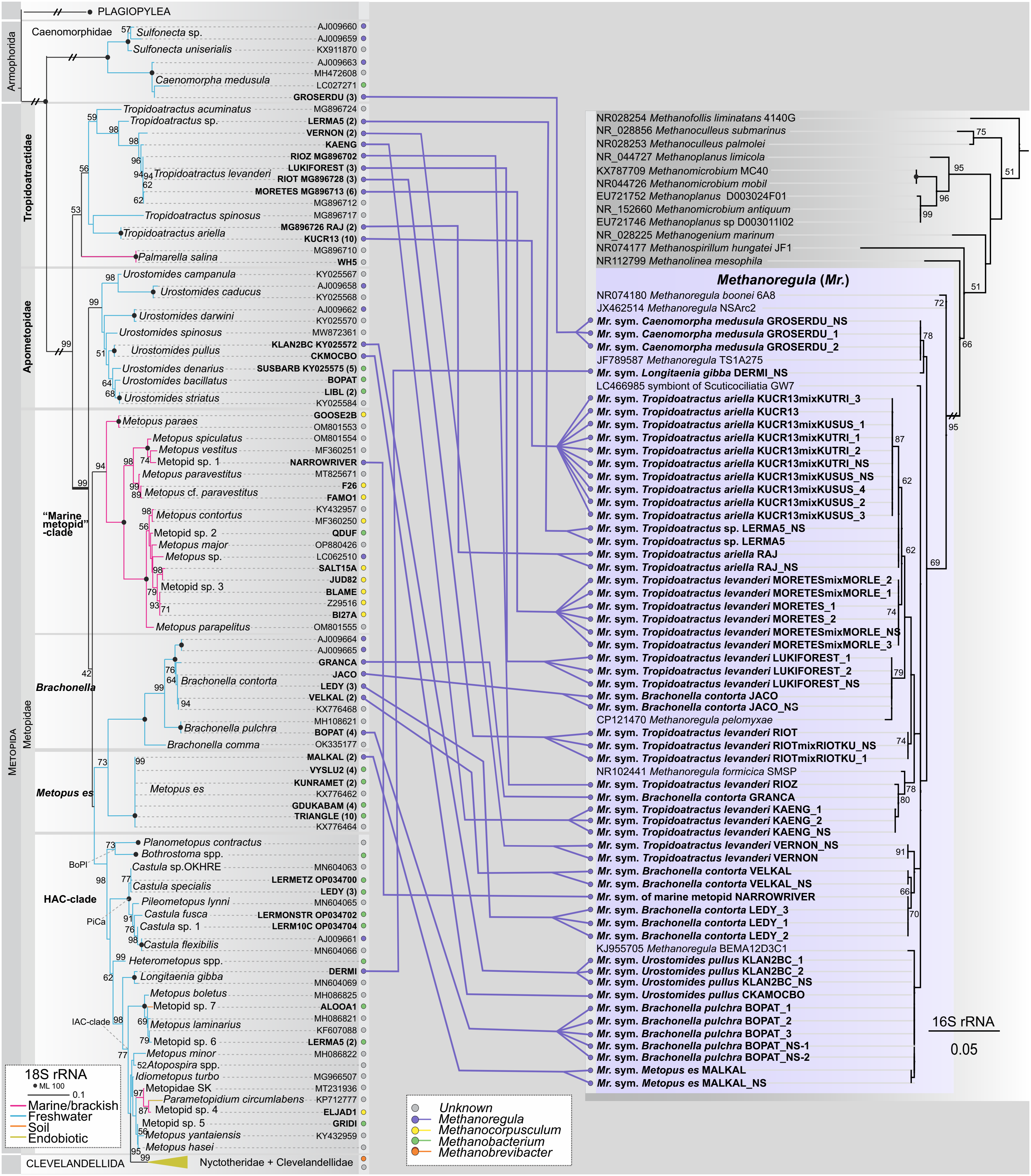

PLAGIOPYLEA
Armophorida
Caenomorphidae
Sulfonecta sp.
Sulfonecta uniserialis
Caenomorpha medusula
AJ009660
AJ009659
KX911870
AJ009663
MH472608
LC027271
GROSERDU (3)
Tropidoatractidae
Tropidoatractus acuminatus
Tropidoatractus sp.
Tropidoatractus levanderi
Tropidoatractus spinosus
Tropidoatractus ariella
Palmarella salina
MG896724
LERMA5 (2)
VERNON (2)
KAENG
RIOZ MG896702
LUKIFOREST (3)
RIOT MG896728 (3)
MORETES MG896713 (6)
MG896712
MG896717
MG896726 RAJ (2)
KUCR13 (10)
MG896710
WH5
Apometopidae
Urostomides campanula
Urostomides caducus
Urostomides darwini
Urostomides spinosus
Urostomides pullus
Urostomides denarius
Urostomides bacillatus
Urostomides striatus
KY025567
AJ009658
KY025568
AJ009662
KY025570
MW872361
KLAN2BC KY025572
CKMOCBO
SUSBARB KY025575 (5)
BOPAT
LIBL (2)
KY025584
"Marine metopid" -clade
Metopus paraes
Metopus spiculatus
Metopus vestitus
Metopid sp. 1
Metopus paravestitus
Metopus cf. paravestitus
Metopus contortus
Metopid sp. 2
Metopus major
Metopus sp.
Metopid sp. 3
Metopus parapelitus
GOOSE2B
OM801553
OM801554
MF360251
NARROWRIVER
MT825671
F26
FAMO1
KY432957
MF360250
QDUF
OP880426
LC062510
SALT15A
JUD82
BLAME
Z29516
BI27A
OM801555
METOPIDA
Metopidae
Brachonella
Brachonella contorta
Brachonella pulchra
Brachonella comma
AJ009664
AJ009665
GRANCA
JACO
LEDY (3)
VELKAL (2)
KX776468
MH108621
BOPAT (4)
OK335177
Metopus es
MALKAL (2)
VYSLU2 (4)
KUNRAMET (2)
KX776462
GDUKABAM (4)
TRIANGLE (10)
KX776464
HAC-clade
BoPl
PiCa
IAC-clade
Planometopus contractus
Bothrostoma spp.
Castula sp.OKHRE
Castula specialis
Pileometopus lynni
Castula fusca
Castula sp. 1
Castula flexibilis
Heterometopus spp.
Longitaenia gibba
Metopus boletus
Metopid sp. 7
Metopus laminarius
Metopid sp. 6
Metopus minor
Atopospira spp.
Idiometopus turbo
Metopidae SK
Parametopidium circumlabens
Metopid sp. 4
Metopid sp. 5
Metopus yantaiensis
Metopus hasei
MN604063
LERMETZ OP034700
LEDY (3)
MN604065
LERMONSTR OP034702
LERM10C OP034704
AJ009661
MN604066
DERMI
MN604069
MH086825
ALOOA1
MH086821
KF607088
LERMA5 (2)
MH086822
MG966507
MT231936
KP712777
ELJAD1
GRIDI
KY432959
CLEVELANDELLIDA
Nyctotheridae + Clevelandellidae
18S rRNA
ML 100
0.1
Marine/brackish
Freshwater
Soil
Endobiotic
Unknown
Methanoregula
Methanocorpusculum
Methanobacterium
Methanobrevibacter
NR028254 Methanofollis liminatans 4140G
NR_028856 Methanoculleus submarinus
NR028253 Methanoculleus palmolei
NR_044727 Methanoplanus limicola
KX787709 Methanomicrobium MC40
NR044726 Methanomicrobium mobil
EU721752 Methanoplanus D003024F01
NR_152660 Methanomicrobium antiquum
EU721746 Methanoplanus sp D003011I02
NR_028225 Methanogenium marinum
NR074177 Methanospirillum hungatei JF1
NR112799 Methanolinea mesophila
Methanoregula (Mr.)
NR074180 Methanoregula boonei 6A8
JX462514 Methanoregula NSArc2
Mr. sym. Caenomorpha medusula GROSERDU_NS
Mr. sym. Caenomorpha medusula GROSERDU_1
Mr. sym. Caenomorpha medusula GROSERDU_2
JF789587 Methanoregula TS1A275
Mr. sym. Longitaenia gibba DERMI_NS
LC466985 symbiont of Scuticociliatia GW7
Mr. sym. Tropidoatractus ariella KUCR13mixKUTRI_3
Mr. sym. Tropidoatractus ariella KUCR13
Mr. sym. Tropidoatractus ariella KUCR13mixKUSUS_1
Mr. sym. Tropidoatractus ariella KUCR13mixKUTRI_1
Mr. sym. Tropidoatractus ariella KUCR13mixKUTRI_2
Mr. sym. Tropidoatractus ariella KUCR13mixKUTRI_NS
Mr. sym. Tropidoatractus ariella KUCR13mixKUSUS_NS
Mr. sym. Tropidoatractus ariella KUCR13mixKUSUS_4
Mr. sym. Tropidoatractus ariella KUCR13mixKUSUS_2
Mr. sym. Tropidoatractus ariella KUCR13mixKUSUS_3
Mr. sym. Tropidoatractus sp. LERMA5_NS
Mr. sym. Tropidoatractus sp. LERMA5
Mr. sym. Tropidoatractus ariella RAJ
Mr. sym. Tropidoatractus ariella RAJ_NS
Mr. sym. Tropidoatractus levanderi MORETESmixMORLE_2
Mr. sym. Tropidoatractus levanderi MORETESmixMORLE_1
Mr. sym. Tropidoatractus levanderi MORETES_1
Mr. sym. Tropidoatractus levanderi MORETES_2
Mr. sym. Tropidoatractus levanderi MORETESmixMORLE_NS
Mr. sym. Tropidoatractus levanderi MORETESmixMORLE_3
Mr. sym. Tropidoatractus levanderi LUKIFOREST_1
Mr. sym. Tropidoatractus levanderi LUKIFOREST_2
Mr. sym. Tropidoatractus levanderi LUKIFOREST_NS
Mr. sym. Brachonella contorta JACO
Mr. sym. Brachonella contorta JACO_NS
CP121470 Methanoregula pelomyxae
Mr. sym. Tropidoatractus levanderi RIOT
Mr. sym. Tropidoatractus levanderi RIOTmixRIOTKU_NS
Mr. sym. Tropidoatractus levanderi RIOTmixRIOTKU_1
NR102441 Methanoregula formicica SMSP
Mr. sym. Tropidoatractus levanderi RIOZ
Mr. sym. Brachonella contorta GRANCA
Mr. sym. Tropidoatractus levanderi KAENG_1
Mr. sym. Tropidoatractus levanderi KAENG_2
Mr. sym. Tropidoatractus levanderi KAENG_NS
Mr. sym. Tropidoatractus levanderi VERNON_NS
Mr. sym. Tropidoatractus levanderi VERNON
Mr. sym. Brachonella contorta VELKAL
Mr. sym. Brachonella contorta VELKAL_NS
Mr. sym. of marine metopid NARROWRIVER
Mr. sym. Brachonella contorta LEDY_3
Mr. sym. Brachonella contorta LEDY_1
Mr. sym. Brachonella contorta LEDY_2
KJ955705 Methanoregula BEMA12D3C1
Mr. sym. Urostomides pullus KLAN2BC_1
Mr. sym. Urostomides pullus KLAN2BC_2
Mr. sym. Urostomides pullus KLAN2BC_NS
Mr. sym. Urostomides pullus CKAMOCBO
Mr. sym. Brachonella pulchra BOPAT_1
Mr. sym. Brachonella pulchra BOPAT_2
Mr. sym. Brachonella pulchra BOPAT_3
Mr. sym. Brachonella pulchra BOPAT_NS-1
Mr. sym. Brachonella pulchra BOPAT_NS-2
Mr. sym. Metopus es MALKAL
Mr. sym. Metopus es MALKAL_NS
16S rRNA
0.05

Figure S3. Maximum likelihood phylogenetic trees based on 18S (left) and 16S (right) rRNA gene sequences showing the connection between ciliate hosts and all obtained symbiotic Sanger sequences belonging to the genus *Methanoregula* (in bold). The habitat of the ciliate is depicted in the 18S tree. The scale bar represents 5 substitutions per 100 positions. Bootstrap values below 50 are not shown.
