# Supplementary material for: Methanogenic symbionts of anaerobic ciliates are host and habitat specific
Source: ISME J. 2024 Aug 20;18(1):wrae164. doi: 10.1093/ismejo/wrae164 (PMC11378729; doi:10.1093/ismejo/wrae164)
Supplement: Supplementary_material [file supplementary_material.zip › FigureS4_MethanocorpusculumTree.pdf]

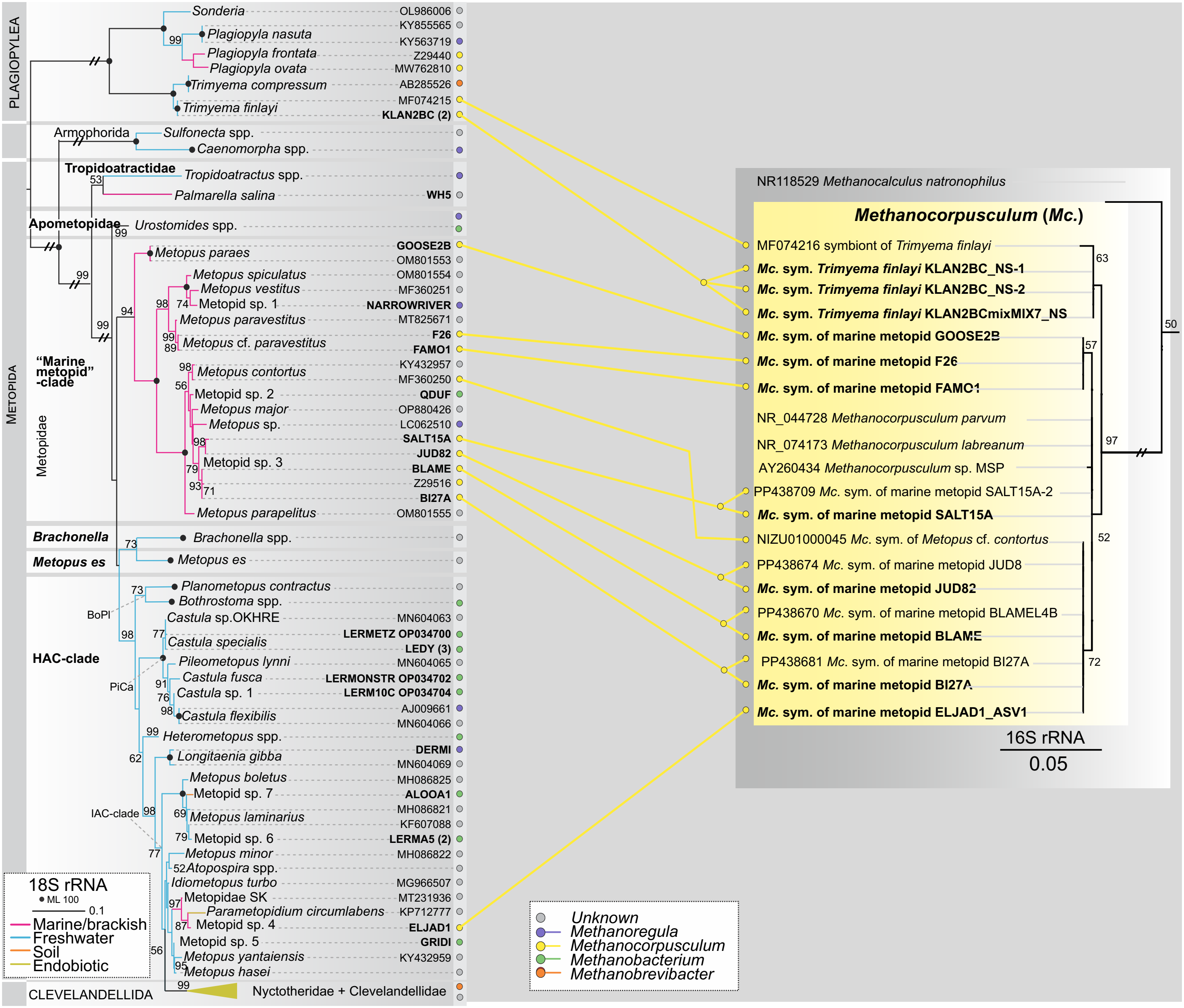
PLAGIOPYLEA
Sonderia OL986006
Plagiopyla nasuta KY855565
KY563719
Plagiopyla frontata Z29440
Plagiopyla ovata MW762810
Trimyema compressum AB285526
Trimyema finlayi MF074215
KLAN2BC (2)
Armophorida
Sulfonecta spp.
Caenomorpha spp.
Tropidoatractidae
Tropidoatractus spp.
Palmarella salina WH5
Apometopidae
Urostomides spp.
Metopus paraes GOOSE2B
OM801553
Metopus spiculatus OM801554
Metopus vestitus MF360251
Metopid sp. 1 NARROWRIVER
Metopus paravestitus MT825671
F26
Metopus cf. paravestitus FAMO1
Metopus contortus KY432957
MF360250
Metopid sp. 2 QDUF
Metopus major OP880426
Metopus sp. LC062510
SALT15A
JUD82
Metopid sp. 3 BLAME
Z29516
BI27A
Metopus parapelitus OM801555
"Marine metopid"-clade
Metopidae
METOPIDA
Brachonella
Brachonella spp.
Metopus es
Metopus es
Planometopus contractus
Bothrostoma spp.
BoPl
Castula sp.OKHRE MN604063
Castula specialis LERMETZ OP034700
LEDY (3)
HAC-clade
Pileometopus lynni MN604065
PiCa
Castula fusca LERMONSTR OP034702
Castula sp. 1 LERM10C OP034704
Castula flexibilis AJ009661
MN604066
Heterometopus spp.
Longitaenia gibba DERMI
MN604069
Metopus boletus MH086825
Metopid sp. 7 ALOOA1
IAC-clade
Metopus laminarius MH086821
KF607088
Metopid sp. 6 LERMA5 (2)
Metopus minor MH086822
Atopospira spp.
Idiometopus turbo MG966507
Metopidae SK MT231936
Parametopidium circumlabens KP712777
Metopid sp. 4 ELJAD1
Metopid sp. 5 GRIDI
Metopus yantaiensis KY432959
Metopus hasei
CLEVELANDELLIDA
Nyctotheridae + Clevelandellidae
18S rRNA
ML 100
0.1
Marine/brackish
Freshwater
Soil
Endobiotic
Unknown
Methanoregula
Methanocorpusculum
Methanobacterium
Methanobrevibacter
NR118529 Methanocalculus natronophilus
Methanocorpusculum (Mc.)
MF074216 symbiont of Trimyema finlayi
Mc. sym. Trimyema finlayi KLAN2BC_NS-1
Mc. sym. Trimyema finlayi KLAN2BC_NS-2
Mc. sym. Trimyema finlayi KLAN2BCmixMIX7_NS
Mc. sym. of marine metopid GOOSE2B
Mc. sym. of marine metopid F26
Mc. sym. of marine metopid FAMO1
NR_044728 Methanocorpusculum parvum
NR_074173 Methanocorpusculum labreanum
AY260434 Methanocorpusculum sp. MSP
PP438709 Mc. sym. of marine metopid SALT15A-2
Mc. sym. of marine metopid SALT15A
NIZU01000045 Mc. sym. of Metopus cf. contortus
PP438674 Mc. sym. of marine metopid JUD8
Mc. sym. of marine metopid JUD82
PP438670 Mc. sym. of marine metopid BLAMEL4B
Mc. sym. of marine metopid BLAME
PP438681 Mc. sym. of marine metopid BI27A
Mc. sym. of marine metopid BI27A
Mc. sym. of marine metopid ELJAD1_ASV1
16S rRNA
0.05

Figure S4. Maximum likelihood phylogenetic trees based on 18S (left) and 16S (right) rRNA gene sequences showing the connection between ciliate hosts and all obtained symbiotic Sanger sequences belonging to the genus *Methanocorpusculum* (in bold). The habitat of the ciliate is depicted in the 18S tree. The scale bar represents 5 substitutions per 100 positions. Bootstrap values below 50 are not shown.
