# Supplementary material for: Methanogenic symbionts of anaerobic ciliates are host and habitat specific
Source: ISME J. 2024 Aug 20;18(1):wrae164. doi: 10.1093/ismejo/wrae164 (PMC11378729; doi:10.1093/ismejo/wrae164)
Supplement: Supplementary_material [file supplementary_material.zip › FigureS5_Barplot.pdf]

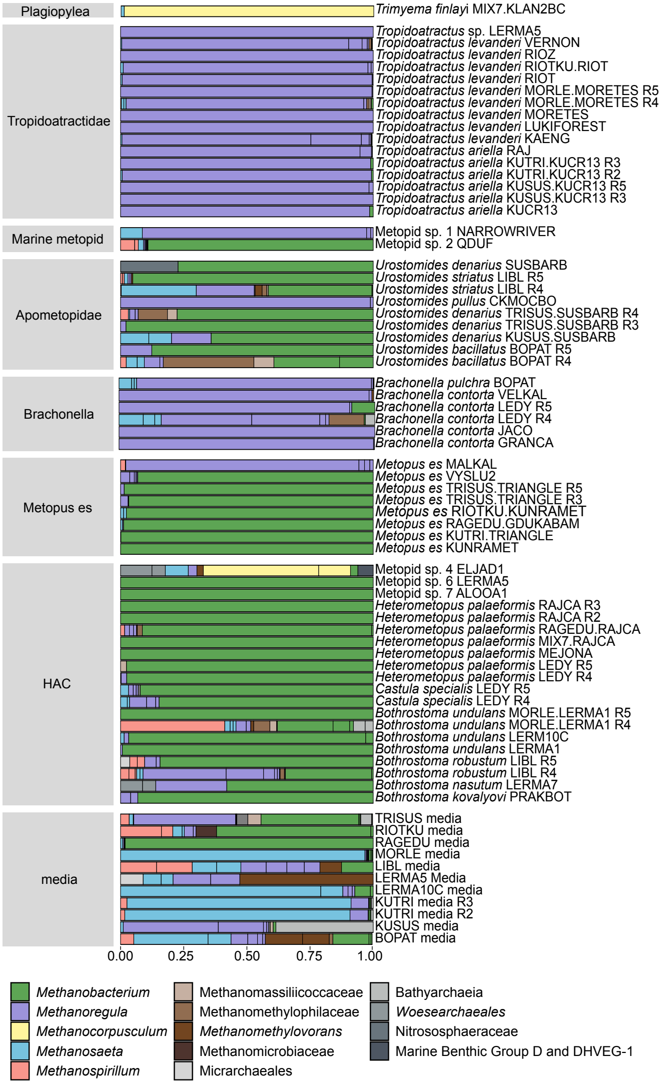

Plagiopylea
Trimyema finlayi MIX7.KLAN2BC
Tropidoatractidae
Tropidoatractus sp. LERMA5
Tropidoatractus levanderi VERNON
Tropidoatractus levanderi RIOZ
Tropidoatractus levanderi RIOTKU.RIOT
Tropidoatractus levanderi RIOT
Tropidoatractus levanderi MORLE.MORETES R5
Tropidoatractus levanderi MORLE.MORETES R4
Tropidoatractus levanderi MORETES
Tropidoatractus levanderi LUKIFOREST
Tropidoatractus levanderi KAENG
Tropidoatractus ariella RAJ
Tropidoatractus ariella KUTRI.KUCR13 R3
Tropidoatractus ariella KUTRI.KUCR13 R2
Tropidoatractus ariella KUSUS.KUCR13 R5
Tropidoatractus ariella KUSUS.KUCR13 R3
Tropidoatractus ariella KUCR13
Marine metopid
Metopid sp. 1 NARROWRIVER
Metopid sp. 2 QDUF
Apometopidae
Urostomides denarius SUSBARB
Urostomides striatus LIBL R5
Urostomides striatus LIBL R4
Urostomides pullus CKMOCBO
Urostomides denarius TRISUS.SUSBARB R4
Urostomides denarius TRISUS.SUSBARB R3
Urostomides denarius KUSUS.SUSBARB
Urostomides bacillatus BOPAT R5
Urostomides bacillatus BOPAT R4
Brachonella
Brachonella pulchra BOPAT
Brachonella contorta VELKAL
Brachonella contorta LEDY R5
Brachonella contorta LEDY R4
Brachonella contorta JACO
Brachonella contorta GRANCA
Metopus es
Metopus es MALKAL
Metopus es VYSLU2
Metopus es TRISUS.TRIANGLE R5
Metopus es TRISUS.TRIANGLE R3
Metopus es RIOTKU.KUNRAMET
Metopus es RAGEDU.GDUKABAM
Metopus es KUTRI.TRIANGLE
Metopus es KUNRAMET
HAC
Metopid sp. 4 ELJAD1
Metopid sp. 6 LERMA5
Metopid sp. 7 ALOOA1
Heterometopus palaeformis RAJCA R3
Heterometopus palaeformis RAJCA R2
Heterometopus palaeformis RAGEDU.RAJCA
Heterometopus palaeformis MIX7.RAJCA
Heterometopus palaeformis MEJONA
Heterometopus palaeformis LEDY R5
Heterometopus palaeformis LEDY R4
Castula specialis LEDY R5
Castula specialis LEDY R4
Bothrostoma undulans MORLE.LERMA1 R5
Bothrostoma undulans MORLE.LERMA1 R4
Bothrostoma undulans LERM10C
Bothrostoma undulans LERMA1
Bothrostoma robustum LIBL R5
Bothrostoma robustum LIBL R4
Bothrostoma nasutum LERMA7
Bothrostoma kovalyovi PRAKBOT
media
TRISUS media
RIOTKU media
RAGEDU media
MORLE media
LIBL media
LERMA5 Media
LERMA10C media
KUTRI media R3
KUTRI media R2
KUSUS media
BOPAT media
0.00
0.25
0.50
0.75
1.00
Methanobacterium
Methanoregula
Methanocorpusculum
Methanosaeta
Methanospirillum
Methanomassiliicoccaceae
Methanomethylophilaceae
Methanomethylovorans
Methanomicrobiaceae
Micrarchaeales
Bathyarchaeia
Woesearchaeales
Nitrososphaeraceae
Marine Benthic Group D and DHVEG-1

Figure S5. Barplot showing the relative abundances of the archaeal genera recovered from 16S rRNA gene amplicon sequencing from each ciliate sample and from the media. Samples are grouped according to the host clade.
