# Supplementary material for: Methanogenic symbionts of anaerobic ciliates are host and habitat specific
Source: ISME J. 2024 Aug 20;18(1):wrae164. doi: 10.1093/ismejo/wrae164 (PMC11378729; doi:10.1093/ismejo/wrae164)
Supplement: Supplementary_material [file supplementary_material.zip › FigureS6_Fulltress.pdf]

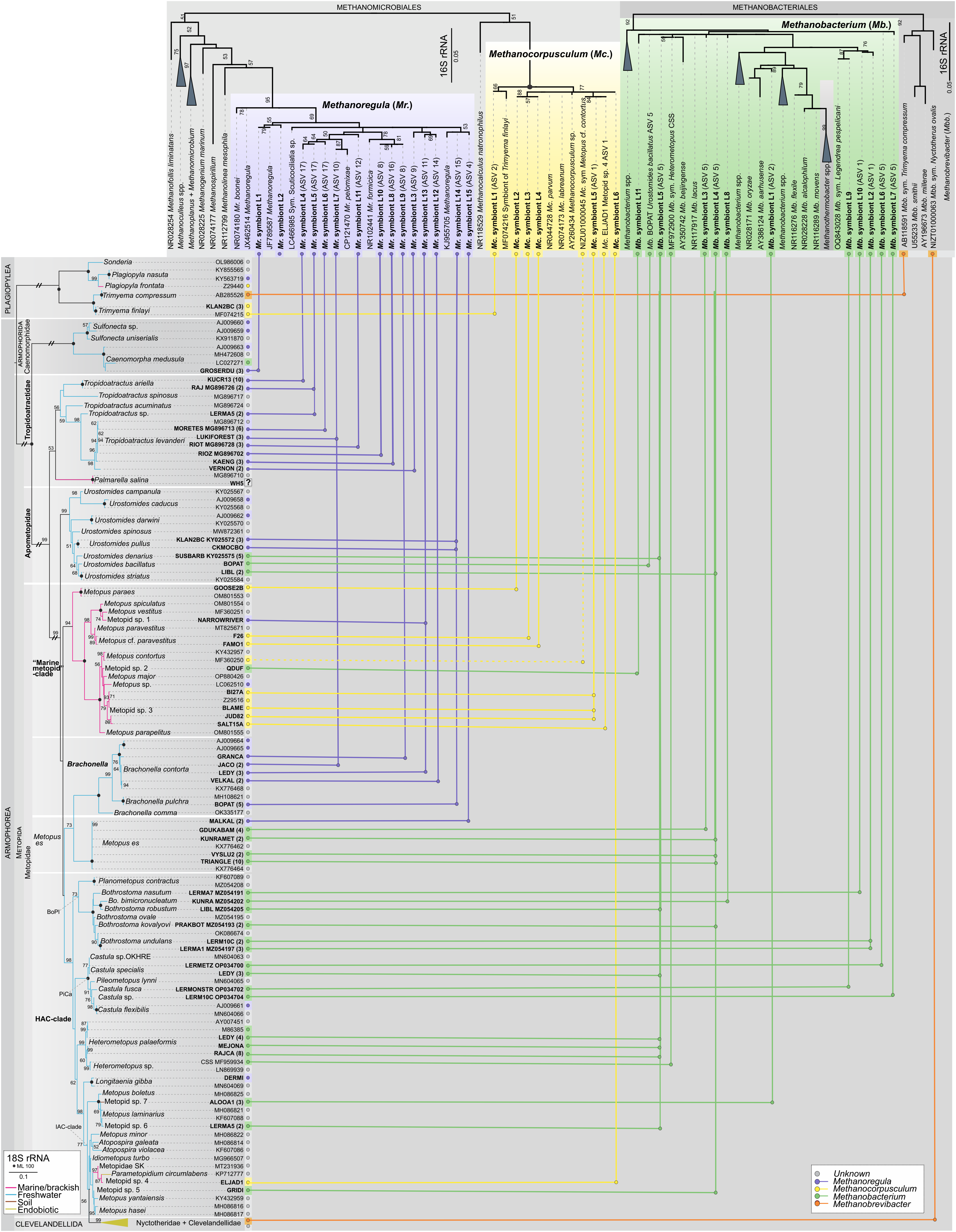
METHANOMICROBIALES
METHANOBACTERIALES
Methanobacterium (Mb.)
Methanocorpusculum (Mc.)
Methanoregula (Mr.)
16S rRNA
0.05
NR028254 Methanofollis liminatans
Methanoculleus spp.
Methanoplanus + Methanomicrobium
NR028225 Methanogenium marinum
NR074177 Methanospirillum
NR112799 Methanolinea mesophila
NR074180 Mr. boonei
JX462514 Methanoregula
Mr. symbiont L1
JF789587 Methanoregula
Mr. symbiont L2
LC466985 Sym. Scuticociliatia sp.
Mr. symbiont L4 (ASV 17)
Mr. symbiont L5 (ASV 17)
Mr. symbiont L6 (ASV 17)
Mr. symbiont L7 (ASV 10)
CP121470 Mr. pelomixae
Mr. symbiont L11 (ASV 12)
NR102441 Mr. formicica
Mr. symbiont L10 (ASV 8)
Mr. symbiont L8 (ASV 16)
Mr. symbiont L9 (ASV 8)
Mr. symbiont L3 (ASV 9)
Mr. symbiont L13 (ASV 11)
Mr. symbiont L12 (ASV 14)
KJ955705 Methanoregula
Mr. symbiont L14 (ASV 15)
Mr. symbiont L15 (ASV 4)
NR118529 Methanocalculus natronophilus
Mc. symbiont L1 (ASV 2)
MF074216 Symbiont of Trimyema finlayi
Mc. symbiont L2
Mc. symbiont L3
Mc. symbiont L4
NR044728 Mc. parvum
NR074173 Mc. labreanum
AY260434 Methanocorpusculum sp.
NIZU01000045 Mc. sym Metopus cf. contortus
Mc. symbiont L5 (ASV 1)
Mc. ELJAD1 Metopid sp. 4 ASV 1
Mc. symbiont L6
Methanobacterium spp.
Mb. symbiont L11
Mb. BOPAT Urostomides bacillatus ASV 5
Mb. symbiont L5 (ASV 5)
MF972900 Mb. sym. Heterometopus CSS
AY350742 Mb. beijingense
NR117917 Mb. lacus
Mb. symbiont L3 (ASV 5)
Mb. symbiont L4 (ASV 5)
Mb. symbiont L8
Methanobacterium spp.
NR028171 Mb. oryzae
AY386124 Mb. aarhusense
Mb. symbiont L1 (ASV 2)
Methanobacterium spp.
NR116276 Mb. flexile
NR028228 Mb. alcaliphilum
NR116289 Mb. movens
Methanothermobacter spp.
OQ843028 Mb. sym. Legendrea pespelicani
Mb. symbiont L9
Mb. symbiont L10 (ASV 1)
Mb. symbiont L2 (ASV 1)
Mb. symbiont L6 (ASV 5)
Mb. symbiont L7 (ASV 5)
AB118591 Mbb. sym. Trimyema compressum
U55233 Mbb. smithii
AY196673 Mbb. millerae
NIZT01000063 Mbb. sym. Nyctotherus ovalis
Methanobrevibacter (Mbb.)
PLAGIOPYLEA
Sonderia OL986006
KY855565
Plagiopyla nasuta KY563719
Plagiopyla frontata Z29440
Trimyema compressum AB285526
KLAN2BC (3)
Trimyema finlayi MF074215
ARMOPHORIDA
Caenomorphidae
Sulfonecta sp. AJ009660
AJ009659
Sulfonecta uniserialis KX911870
AJ009663
MH472608
Caenomorpha medusula LC027271
GROSERDU (3)
Tropidoatractidae
Tropidoatractus ariella KUCR13 (10)
RAJ MG896726 (2)
Tropidoatractus spinosus MG896717
Tropidoatractus acuminatus MG896724
Tropidoatractus sp. LERMA5 (2)
MG896712
MORETES MG896713 (6)
LUKIFOREST (3)
Tropidoatractus levanderi RIOT MG896728 (3)
RIOZ MG896702
KAENG (3)
VERNON (2)
MG896710
Palmarella salina WH5 ?
Apometopidae
Urostomides campanula KY025567
Urostomides caducus AJ009658
KY025568
Urostomides darwini AJ009662
KY025570
Urostomides spinosus MW872361
Urostomides pullus KLAN2BC KY025572 (3)
CKMOCBO
Urostomides denarius SUSBARB KY025575 (5)
Urostomides bacillatus BOPAT
Urostomides striatus LIBL (2)
KY025584
"Marine metopid" -clade
Metopus paraes GOOSE2B
OM801553
Metopus spiculatus OM801554
Metopus vestitus MF360251
Metopid sp. 1 NARROWRIVER
Metopus paravestitus MT825671
F26
Metopus cf. paravestitus FAMO1
Metopus contortus KY432957
MF360250
Metopid sp. 2 QDUF
Metopus major OP880426
Metopus sp. LC062510
BI27A
Z29516
Metopid sp. 3 BLAME
JUD82
SALT15A
Metopus parapelitus OM801555
Brachonella
AJ009664
AJ009665
GRANCA
JACO (2)
Brachonella contorta LEDY (3)
VELKAL (2)
KX776468
MH108621
Brachonella pulchra BOPAT (5)
Brachonella comma OK335177
MALKAL (2)
Metopus es
GDUKABAM (4)
KUNRAMET (2)
Metopus es KX776462
VYSLU2 (2)
TRIANGLE (10)
KX776464
Planometopus contractus KF607089
MZ054208
BoPl
Bothrostoma nasutum LERMA7 MZ054191
Bo. bimicronucleatum KUNRA MZ054202
Bothrostoma robustum LIBL MZ054205
Bothrostoma ovale MZ054195
Bothrostoma kovalyovi PRAKBOT MZ054193 (2)
OK086674
Bothrostoma undulans LERM10C (2)
LERMA1 MZ054197 (3)
Castula sp.OKHRE MN604063
LERMETZ OP034700
Castula specialis LEDY (3)
Pileometopus lynni MN604065
PiCa
Castula fusca LERMONSTR OP034702
Castula sp. LERM10C OP034704
AJ009661
Castula flexibilis MN604066
HAC-clade
AY007451
M86385
Heterometopus palaeformis LEDY (4)
MEJONA
RAJCA (8)
CSS MF959934
Heterometopus sp. LN869939
Longitaenia gibba DERMI
MN604069
Metopus boletus MH086825
Metopid sp. 7 ALOOA1 (3)
MH086821
Metopus laminarius KF607088
Metopid sp. 6 LERMA5 (2)
IAC-clade
Metopus minor MH086822
Atopospira galeata MH086814
Atopospira violacea KF607086
Idiometopus turbo MG966507
Metopidae SK MT231936
Parametopidium circumlabens KP712777
Metopid sp. 4 ELJAD1
Metopid sp. 5 GRIDI
Metopus yantaiensis KY432959
MH086816
Metopus hasei MH086817
ARMOPHOREA
METOPIDA
Metopidae
CLEVELANDELLIDA
Nyctotheridae + Clevelandellidae
18S rRNA
ML 100
0.1
Marine/brackish
Freshwater
Soil
Endobiotic
Unknown
Methanoregula
Methanocorpusculum
Methanobacterium
Methanobrevibacter

Figure S6. Maximum likelihood phylogenetic trees based on 18S (left) and 16S (right) rRNA gene sequences showing the connection between ciliate hosts and their respective methanogenic symbionts. The habitat of the ciliate is depicted in the 18S tree. The number of 16S rRNA Sanger sequences obtained per ciliate strain is in brackets. The corresponding dominant ASVs which are 100% identical to the symbiont lineages are indicated in the 16S tree in brackets. Bootstrap values below 50 are not shown. The scale bar represents 10 substitutions per 100 positions in the 18S tree and 5 substitutions per 100 positions in the 16S trees.
