# Supplementary material for: Methanogenic symbionts of anaerobic ciliates are host and habitat specific
Source: ISME J. 2024 Aug 20;18(1):wrae164. doi: 10.1093/ismejo/wrae164 (PMC11378729; doi:10.1093/ismejo/wrae164)
Supplement: Supplementary_material [file supplementary_material.zip › FigureS7_CollapsedTrees.pdf]

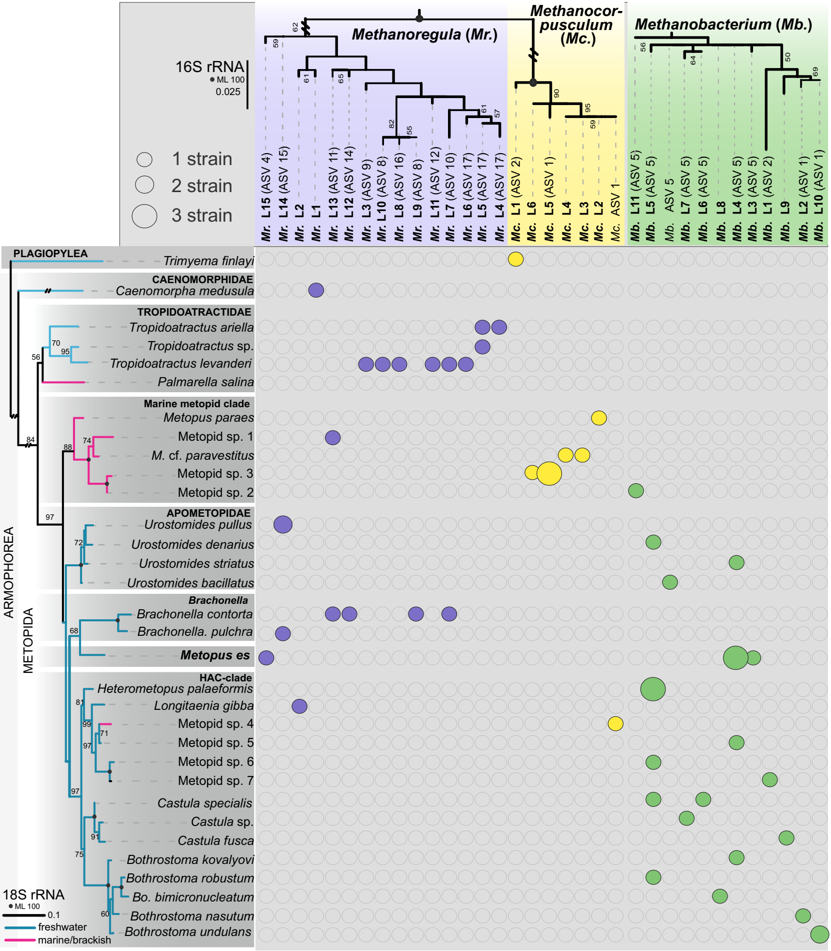

16S rRNA
ML 100
0.025
1 strain
2 strain
3 strain
Methanoregula (Mr.)
Methanocorpusculum (Mc.)
Methanobacterium (Mb.)
Mr. L15 (ASV 4)
Mr. L14 (ASV 15)
Mr. L2
Mr. L1
Mr. L13 (ASV 11)
Mr. L12 (ASV 14)
Mr. L3 (ASV 9)
Mr. L10 (ASV 8)
Mr. L8 (ASV 16)
Mr. L9 (ASV 8)
Mr. L11 (ASV 12)
Mr. L7 (ASV 10)
Mr. L6 (ASV 17)
Mr. L5 (ASV 17)
Mr. L4 (ASV 17)
Mc. L1 (ASV 2)
Mc. L6
Mc. L5 (ASV 1)
Mc. L4
Mc. L3
Mc. L2
Mc. ASV 1
Mb. L11 (ASV 5)
Mb. L5 (ASV 5)
Mb. ASV 5
Mb. L7 (ASV 5)
Mb. L6 (ASV 5)
Mb. L8
Mb. L4 (ASV 5)
Mb. L3 (ASV 5)
Mb. L1 (ASV 2)
Mb. L9
Mb. L2 (ASV 1)
Mb. L10 (ASV 1)
PLAGIOPYLEA
Trimyema finlayi
CAENOMORPHIDAE
Caenomorpha medusula
TROPIDOATRACTIDAE
Tropidoatractus ariella
Tropidoatractus sp.
Tropidoatractus levanderi
Palmarella salina
Marine metopid clade
Metopus paraes
Metopid sp. 1
M. cf. paravestitus
Metopid sp. 3
Metopid sp. 2
APOMETOPIDAE
Urostomides pullus
Urostomides denarius
Urostomides striatus
Urostomides bacillatus
Brachonella
Brachonella contorta
Brachonella. pulchra
Metopus es
HAC-clade
Heterometopus palaeformis
Longitaenia gibba
Metopid sp. 4
Metopid sp. 5
Metopid sp. 6
Metopid sp. 7
Castula specialis
Castula sp.
Castula fusca
Bothrostoma kovalyovi
Bothrostoma robustum
Bo. bimicronucleatum
Bothrostoma nasutum
Bothrostoma undulans
ARMOPHOREA
METOPIDA
18S rRNA
ML 100
0.1
freshwater
marine/brackish

Figure S7. Distribution of the methanogenic symbiont lineages (top tree) across the host ciliate species (left tree). The number of host strains per each ciliate species is represented by bubbles.
