# Supplementary material for: Methanogenic symbionts of anaerobic ciliates are host and habitat specific
Source: ISME J. 2024 Aug 20;18(1):wrae164. doi: 10.1093/ismejo/wrae164 (PMC11378729; doi:10.1093/ismejo/wrae164)
Supplement: Supplementary_material [file supplementary_material.zip › FigureS8_SummaryResults.pdf]

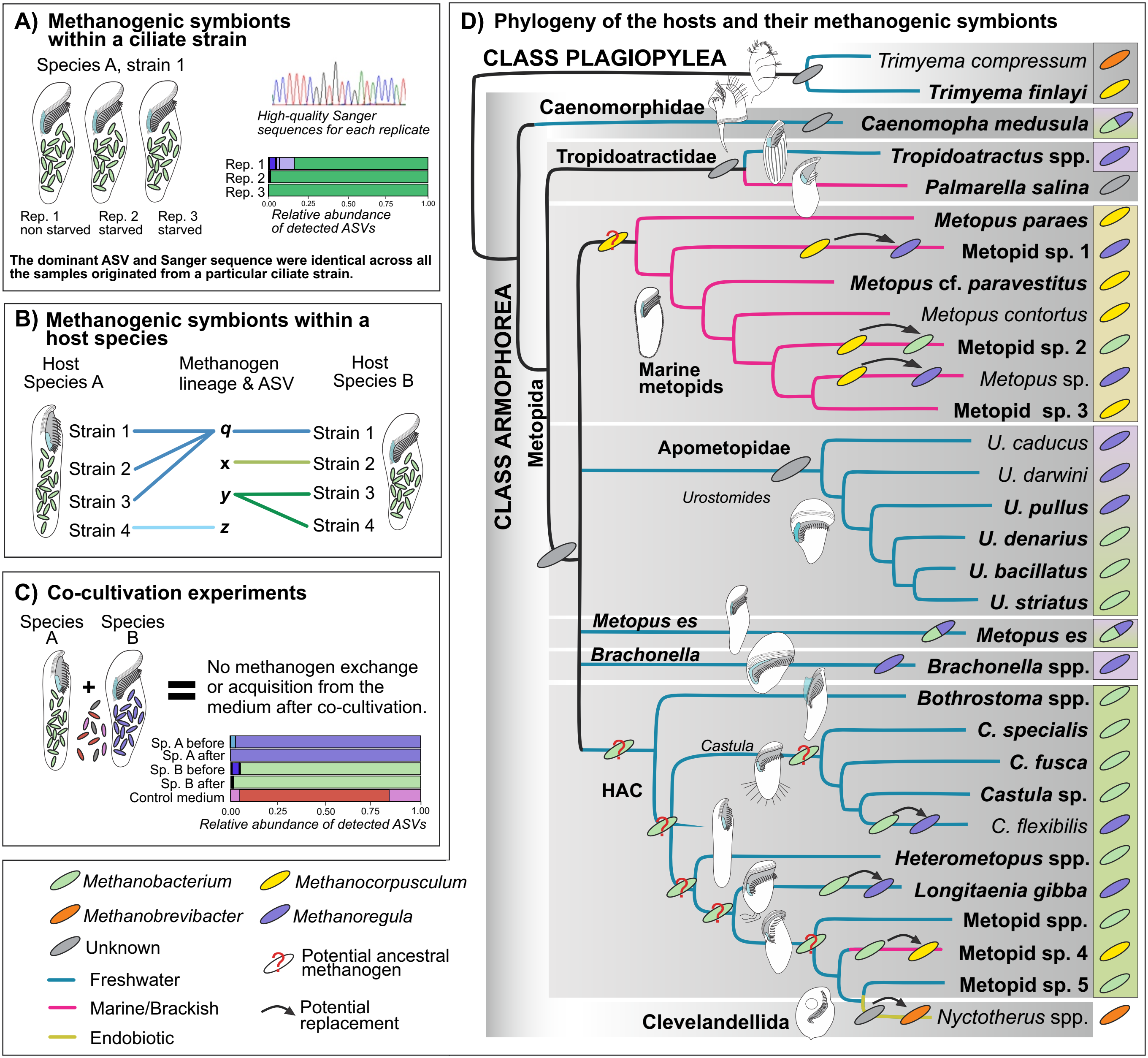
A) Methanogenic symbionts within a ciliate strain
Species A, strain 1
High-quality Sanger sequences for each replicate
Rep. 1
Rep. 2
Rep. 3
0.00
0.25
0.50
0.75
1.00
Relative abundance of detected ASVs
Rep. 1 non starved
Rep. 2 starved
Rep. 3 starved
The dominant ASV and Sanger sequence were identical across all the samples originated from a particular ciliate strain.
B) Methanogenic symbionts within a host species
Host Species A
Methanogen lineage & ASV
Host Species B
Strain 1
Strain 2
Strain 3
Strain 4
q
x
y
z
Strain 1
Strain 2
Strain 3
Strain 4
C) Co-cultivation experiments
Species A
Species B
No methanogen exchange or acquisition from the medium after co-cultivation.
Sp. A before
Sp. A after
Sp. B before
Sp. B after
Control medium
0.00
0.25
0.50
0.75
1.00
Relative abundance of detected ASVs
Methanobacterium
Methanocorpusculum
Methanobrevibacter
Methanoregula
Unknown
Potential ancestral methanogen
Freshwater
Marine/Brackish
Potential replacement
Endobiotic
D) Phylogeny of the hosts and their methanogenic symbionts
CLASS PLAGIOPYLEA
Trimyema compressum
Trimyema finlayi
Caenomorphidae
Caenomopha medusula
Tropidoatractidae
Tropidoatractus spp.
Palmarella salina
CLASS ARMOPHOREA
Metopida
Metopus paraes
Metopid sp. 1
Metopus cf. paravestitus
Metopus contortus
Metopid sp. 2
Metopus sp.
Metopid sp. 3
Marine metopids
Apometopidae
Urostomides
U. caducus
U. darwini
U. pullus
U. denarius
U. bacillatus
U. striatus
Metopus es
Metopus es
Brachonella
Brachonella spp.
Bothrostoma spp.
Castula
C. specialis
C. fusca
Castula sp.
C. flexibilis
HAC
Heterometopus spp.
Longitaenia gibba
Metopid spp.
Metopid sp. 4
Metopid sp. 5
Clevelandellida
Nyctotherus spp.

Figure S8A-D. Summary of results. A, Methanogenic symbionts within a strain are genetically identical across replicates regardless of starved or non-starved conditions. B, Methanogenic symbionts of different strains within a ciliate species (intraspecific) are genetically divergent in most cases. C, Methanogenic symbionts of co-occurring unrelated ciliate species are maintained rather than exchanged, and the archeal communities from the control medium differ from those in the ciliates. D, Schematic phylogenetic tree of the ciliate hosts and their respective methanogenic symbionts. Potential ancestral and replacements of the methanogenic symbionts are shown. Species examined in this study are in bold.
