# Supplementary material for: Methanogenic symbionts of anaerobic ciliates are host and habitat specific
Source: ISME J. 2024 Aug 20;18(1):wrae164. doi: 10.1093/ismejo/wrae164 (PMC11378729; doi:10.1093/ismejo/wrae164)
Supplement: Supplementary_material [file supplementary_material.zip › SupplementalFigures_final_wrae164.pdf]

Methanogenic symbionts of anaerobic ciliates are host- and habitat-specific

Daniel Méndez-Sánchez[1, a, *], Anna Schrecengost[2, a], Johana Rotterová[1, 2, b], Kateřina Koštířová[1], Roxanne A. Beinart[2], Ivan Čepička[1, *]

[1]Department of Zoology, Faculty of Science, Charles University, Viničná 7, 128 00 Prague 2, Czech Republic.

[2]Graduate School of Oceanography, University of Rhode Island, Narragansett, Rhode Island, USA.

[a]Both authors contributed equally to the work.

[b]Current address: Department of Marine Sciences, University of Puerto Rico Mayagüez, Mayagüez, Puerto Rico, USA.

[*]Corresponding authors: Daniel Méndez-Sánchez, Department of Zoology, Faculty of Science, Charles University, Viničná 7, Prague, 128 00 Prague 2, Czech Republic, email: mendezsd@natur.cuni.cz, and Ivan Čepička, Department of Zoology, Faculty of Science, Charles University, Viničná 7, Prague, 128 00 Prague 2, Czech Republic, tel: (+420) 221 951 812, email: ivan.cepicka@natur.cuni.cz

Supplemental figures

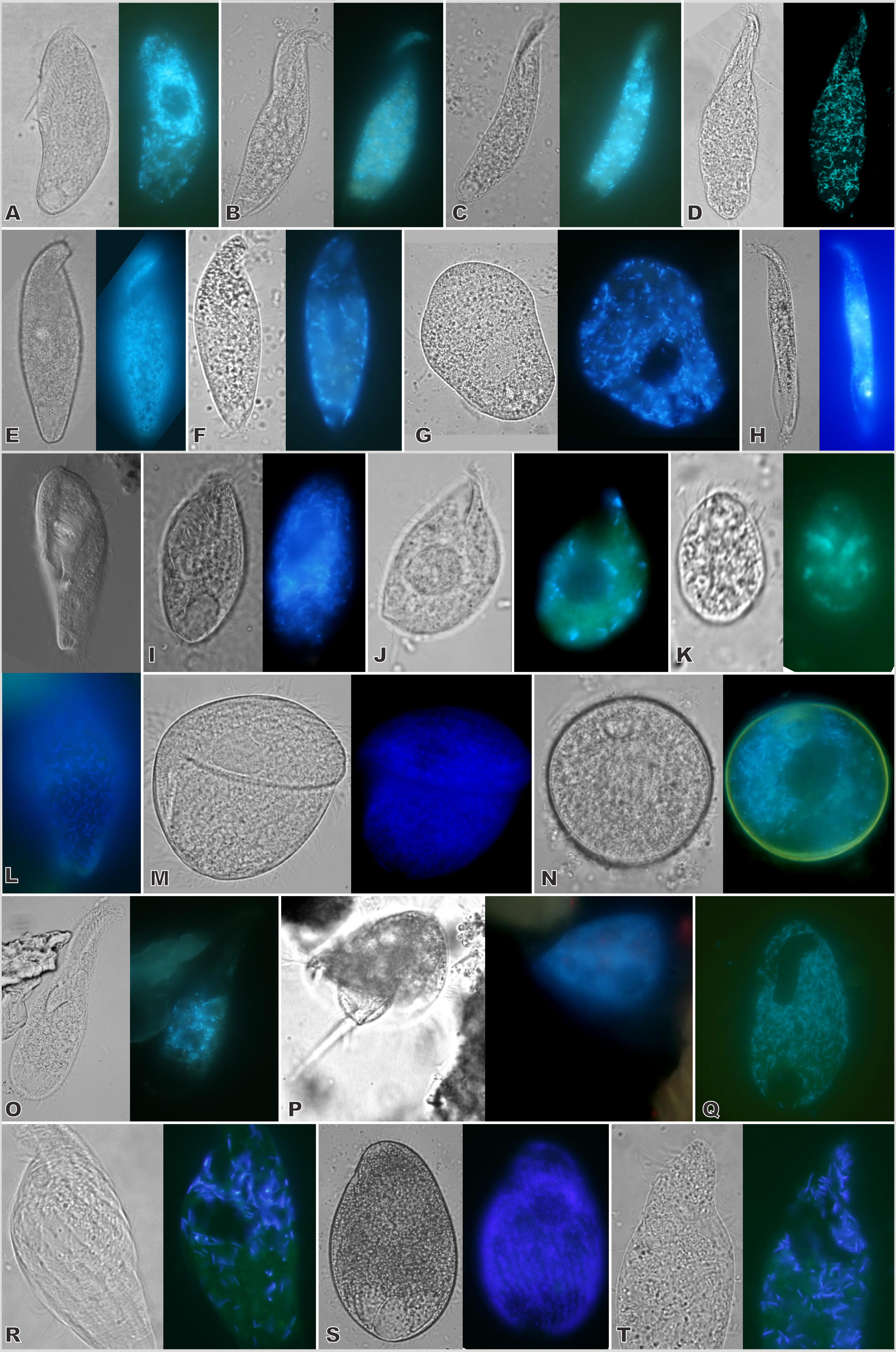
A
B
C
D
E
F
G
H
I
J
K
L
M
N
O
P
Q
R
S
T

Figure S1. Anaerobic ciliates in bright field (left or up) and under autofluorescence (right or down) showing the rod-shaped methanogenic symbionts. D. Confocal microscopy. A, *Bothrosrtoma undulans* LERMA1. B, Metopid sp. 7 ALOOA1. C–D, *Heterometopus palaeformis* RAJCA before (C) and after mixing (D). E, *Metopus es* GDUKABAM. F, *Tropidoatractus ariella* KUCR13. G, *Urostomides denarius* SUSBARB. H, Metopid sp. 4 ELJAD1. I, *Bothrostoma nasutum* LERMA7. J, *Tropidoatractus* sp. LERMA5. K, *Trimyema finlayi* KLAN2BC. L, *Bothrostoma robustum* LIBL. M, *Brachonella pulchra* BOPAT. N, Cyst of *Urostomides bacillatus* BOPAT. O, Metopid sp. 5 LERMA5. P, *Caenomorpha medusula* GROSERDU. Q, *Tropidoatractus levanderi* VERNON. R, *Tropidoatractus ariella* RAJ. S, *Metopus es* TRIANGLE. T, *Heterometopus palaeformis* MEJONA.

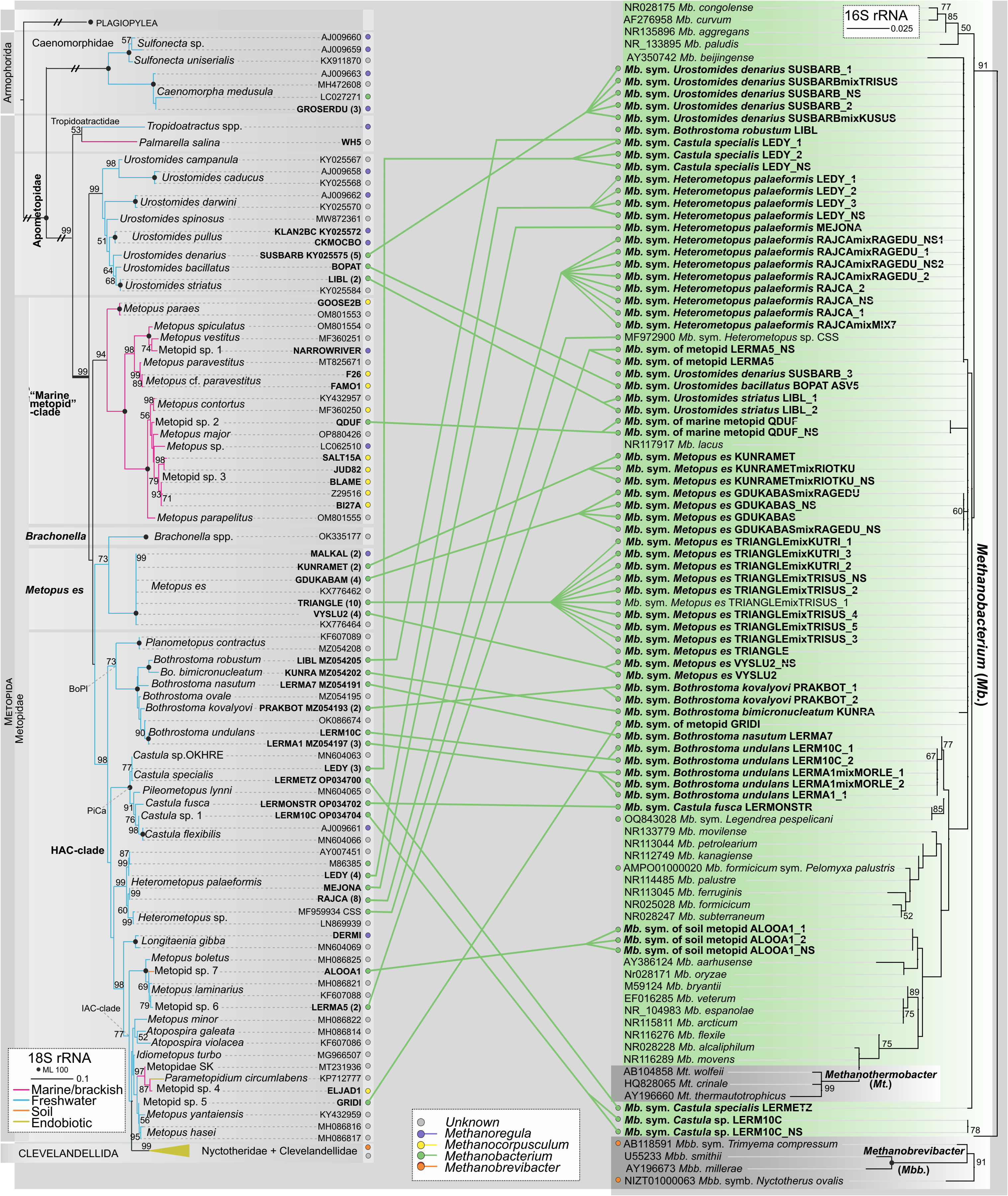

PLAGIOPYLEA
Armophorida
Caenomorphidae
Sulfonecta sp.
Sulfonecta uniserialis
Caenomorpha medusula
AJ009660
AJ009659
KX911870
AJ009663
MH472608
LC027271
GROSERDU (3)
Tropidoatractidae
Tropidoatractus spp.
Palmarella salina
WH5
Apometopidae
Urostomides campanula
Urostomides caducus
Urostomides darwini
Urostomides spinosus
Urostomides pullus
Urostomides denarius
Urostomides bacillatus
Urostomides striatus
KY025567
AJ009658
KY025568
AJ009662
KY025570
MW872361
KLAN2BC KY025572
CKMOCBO
SUSBARB KY025575 (5)
BOPAT
LIBL (2)
KY025584
"Marine metopid" -clade
Metopus paraes
Metopus spiculatus
Metopus vestitus
Metopid sp. 1
Metopus paravestitus
Metopus cf. paravestitus
Metopus contortus
Metopid sp. 2
Metopus major
Metopus sp.
Metopid sp. 3
Metopus parapelitus
GOOSE2B
OM801553
OM801554
MF360251
NARROWRIVER
MT825671
F26
FAMO1
KY432957
MF360250
QDUF
OP880426
LC062510
SALT15A
JUD82
BLAME
Z29516
BI27A
OM801555
Brachonella
Brachonella spp.
OK335177
Metopus es
MALKAL (2)
KUNRAMET (2)
GDUKABAM (4)
KX776462
TRIANGLE (10)
VYSLU2 (4)
KX776464
METOPIDA
Metopidae
BoPl
Planometopus contractus
Bothrostoma robustum
Bo. bimicronucleatum
Bothrostoma nasutum
Bothrostoma ovale
Bothrostoma kovalyovi
Bothrostoma undulans
KF607089
MZ054208
LIBL MZ054205
KUNRA MZ054202
LERMA7 MZ054191
MZ054195
PRAKBOT MZ054193 (2)
OK086674
LERM10C
LERMA1 MZ054197 (3)
PiCa
Castula sp.OKHRE
Castula specialis
Pileometopus lynni
Castula fusca
Castula sp. 1
Castula flexibilis
MN604063
LEDY (3)
LERMETZ OP034700
MN604065
LERMONSTR OP034702
LERM10C OP034704
AJ009661
MN604066
HAC-clade
Heterometopus palaeformis
Heterometopus sp.
AY007451
M86385
LEDY (4)
MEJONA
RAJCA (8)
MF959934 CSS
LN869939
Longitaenia gibba
Metopus boletus
Metopid sp. 7
Metopus laminarius
Metopid sp. 6
IAC-clade
Metopus minor
Atopospira galeata
Atopospira violacea
Idiometopus turbo
Metopidae SK
Parametopidium circumlabens
Metopid sp. 4
Metopid sp. 5
Metopus yantaiensis
Metopus hasei
DERMI
MN604069
MH086825
ALOOA1
MH086821
KF607088
LERMA5 (2)
MH086822
MH086814
KF607086
MG966507
MT231936
KP712777
ELJAD1
GRIDI
KY432959
MH086816
MH086817
CLEVELANDELLIDA
Nyctotheridae + Clevelandellidae
18S rRNA
ML 100
0.1
Marine/brackish
Freshwater
Soil
Endobiotic
Unknown
Methanoregula
Methanocorpusculum
Methanobacterium
Methanobrevibacter
16S rRNA
0.025
NR028175 Mb. congolense
AF276958 Mb. curvum
NR135896 Mb. aggregans
NR_133895 Mb. paludis
AY350742 Mb. beijingense
Mb. sym. Urostomides denarius SUSBARB_1
Mb. sym. Urostomides denarius SUSBARBmixTRISUS
Mb. sym. Urostomides denarius SUSBARB_NS
Mb. sym. Urostomides denarius SUSBARB_2
Mb. sym. Urostomides denarius SUSBARBmixKUSUS
Mb. sym. Bothrostoma robustum LIBL
Mb. sym. Castula specialis LEDY_1
Mb. sym. Castula specialis LEDY_2
Mb. sym. Castula specialis LEDY_NS
Mb. sym. Heterometopus palaeformis LEDY_1
Mb. sym. Heterometopus palaeformis LEDY_2
Mb. sym. Heterometopus palaeformis LEDY_3
Mb. sym. Heterometopus palaeformis LEDY_NS
Mb. sym. Heterometopus palaeformis MEJONA
Mb. sym. Heterometopus palaeformis RAJCAmixRAGEDU_NS1
Mb. sym. Heterometopus palaeformis RAJCAmixRAGEDU_1
Mb. sym. Heterometopus palaeformis RAJCAmixRAGEDU_NS2
Mb. sym. Heterometopus palaeformis RAJCAmixRAGEDU_2
Mb. sym. Heterometopus palaeformis RAJCA_2
Mb. sym. Heterometopus palaeformis RAJCA_NS
Mb. sym. Heterometopus palaeformis RAJCA_1
Mb. sym. Heterometopus palaeformis RAJCAmixMIX7
MF972900 Mb. sym. Heterometopus sp. CSS
Mb. sym. of metopid LERMA5_NS
Mb. sym. of metopid LERMA5
Mb. sym. Urostomides denarius SUSBARB_3
Mb. sym. Urostomides bacillatus BOPAT ASV5
Mb. sym. Urostomides striatus LIBL_1
Mb. sym. Urostomides striatus LIBL_2
Mb. sym. of marine metopid QDUF
Mb. sym. of marine metopid QDUF_NS
NR117917 Mb. lacus
Mb. sym. Metopus es KUNRAMET
Mb. sym. Metopus es KUNRAMETmixRIOTKU
Mb. sym. Metopus es KUNRAMETmixRIOTKU_NS
Mb. sym. Metopus es GDUKABASmixRAGEDU
Mb. sym. Metopus es GDUKABAS_NS
Mb. sym. Metopus es GDUKABAS
Mb. sym. Metopus es GDUKABASmixRAGEDU_NS
Mb. sym. Metopus es TRIANGLEmixKUTRI_1
Mb. sym. Metopus es TRIANGLEmixKUTRI_3
Mb. sym. Metopus es TRIANGLEmixKUTRI_2
Mb. sym. Metopus es TRIANGLEmixTRISUS_NS
Mb. sym. Metopus es TRIANGLEmixTRISUS_2
Mb. sym. Metopus es TRIANGLEmixTRISUS_1
Mb. sym. Metopus es TRIANGLEmixTRISUS_4
Mb. sym. Metopus es TRIANGLEmixTRISUS_5
Mb. sym. Metopus es TRIANGLEmixTRISUS_3
Mb. sym. Metopus es TRIANGLE
Mb. sym. Metopus es VYSLU2_NS
Mb. sym. Metopus es VYSLU2
Mb. sym. Bothrostoma kovalyovi PRAKBOT_1
Mb. sym. Bothrostoma kovalyovi PRAKBOT_2
Mb. sym. Bothrostoma bimicronucleatum KUNRA
Mb. sym. of metopid GRIDI
Mb. sym. Bothrostoma nasutum LERMA7
Mb. sym. Bothrostoma undulans LERM10C_1
Mb. sym. Bothrostoma undulans LERM10C_2
Mb. sym. Bothrostoma undulans LERMA1mixMORLE_1
Mb. sym. Bothrostoma undulans LERMA1mixMORLE_2
Mb. sym. Bothrostoma undulans LERMA1_1
Mb. sym. Castula fusca LERMONSTR
OQ843028 Mb. sym. Legendrea pespelicani
NR133779 Mb. movilense
NR113044 Mb. petrolearium
NR112749 Mb. kanagiense
AMPO01000020 Mb. formicicum sym. Pelomyxa palustris
NR114485 Mb. palustre
NR113045 Mb. ferruginis
NR025028 Mb. formicicum
NR028247 Mb. subterraneum
Mb. sym. of soil metopid ALOOA1_1
Mb. sym. of soil metopid ALOOA1_2
Mb. sym. of soil metopid ALOOA1_NS
AY386124 Mb. aarhusense
Nr028171 Mb. oryzae
M59124 Mb. bryantii
EF016285 Mb. veterum
NR_104983 Mb. espanolae
NR115811 Mb. arcticum
NR116276 Mb. flexile
NR028228 Mb. alcaliphilum
NR116289 Mb. movens
AB104858 Mt. wolfeii
HQ828065 Mt. crinale
AY196660 Mt. thermautotrophicus
Methanothermobacter (Mt.)
Mb. sym. Castula specialis LERMETZ
Mb. sym. Castula sp. LERM10C
Mb. sym. Castula sp. LERM10C_NS
Methanobacterium (Mb.)
AB118591 Mbb. sym. Trimyema compressum
U55233 Mbb. smithii
AY196673 Mbb. millerae
NIZT01000063 Mbb. symb. Nyctotherus ovalis
Methanobrevibacter (Mbb.)

Figure S2. Maximum likelihood phylogenetic trees based on 18S (left) and 16S (right) rRNA gene sequences showing the connection between ciliate hosts and all obtained symbiotic Sanger sequences belonging to the genus *Methanobacterium* (in bold). The habitat of the ciliate is depicted in the 18S tree. The scale bar represents 5 substitutions per 100 positions. Bootstrap values below 50 are not shown.

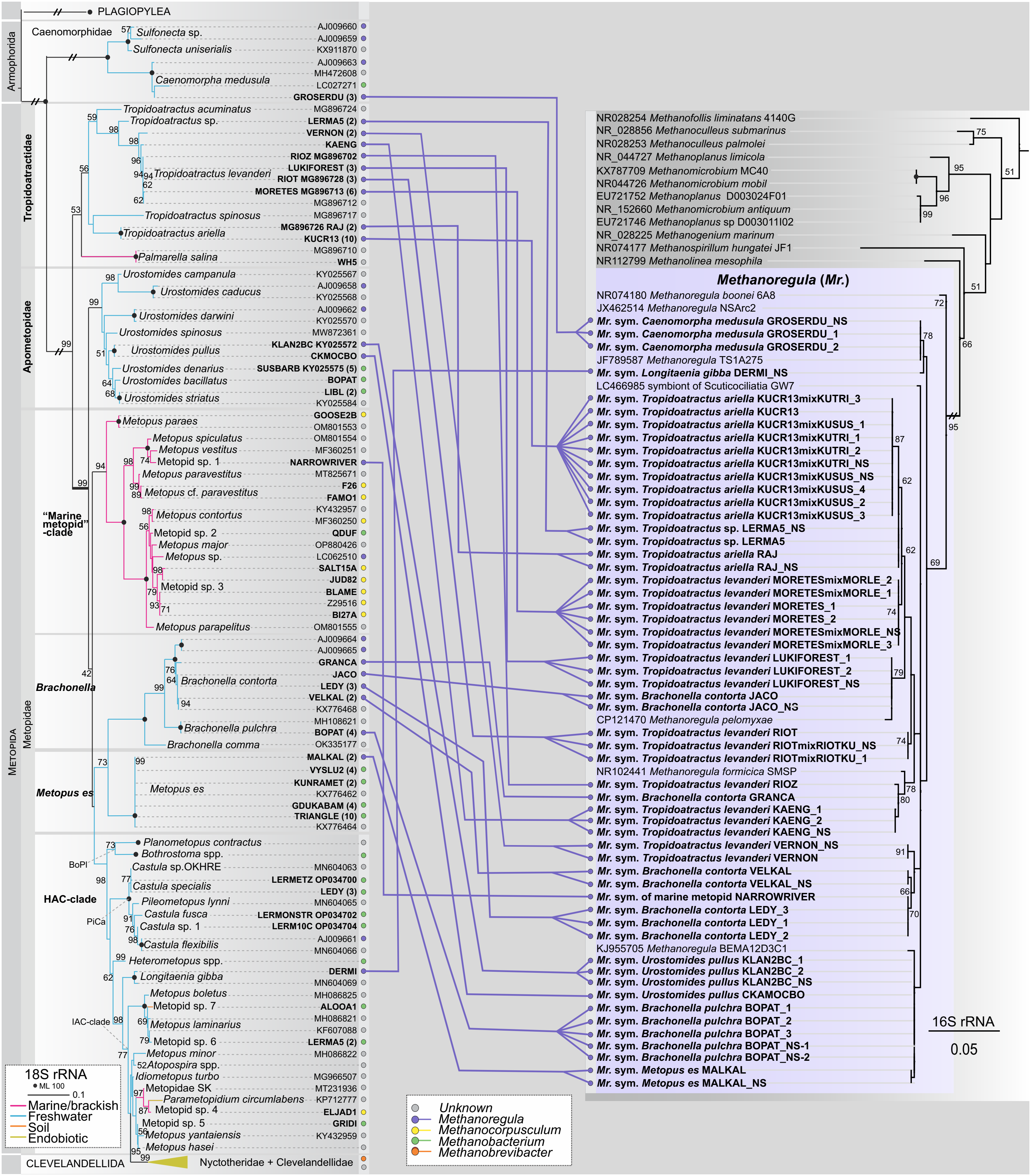

PLAGIOPYLEA
Armophorida
Caenomorphidae
Sulfonecta sp.
Sulfonecta uniserialis
Caenomorpha medusula
AJ009660
AJ009659
KX911870
AJ009663
MH472608
LC027271
GROSERDU (3)
Tropidoatractidae
Tropidoatractus acuminatus
Tropidoatractus sp.
Tropidoatractus levanderi
Tropidoatractus spinosus
Tropidoatractus ariella
Palmarella salina
MG896724
LERMA5 (2)
VERNON (2)
KAENG
RIOZ MG896702
LUKIFOREST (3)
RIOT MG896728 (3)
MORETES MG896713 (6)
MG896712
MG896717
MG896726 RAJ (2)
KUCR13 (10)
MG896710
WH5
Apometopidae
Urostomides campanula
Urostomides caducus
Urostomides darwini
Urostomides spinosus
Urostomides pullus
Urostomides denarius
Urostomides bacillatus
Urostomides striatus
KY025567
AJ009658
KY025568
AJ009662
KY025570
MW872361
KLAN2BC KY025572
CKMOCBO
SUSBARB KY025575 (5)
BOPAT
LIBL (2)
KY025584
"Marine metopid" -clade
Metopus paraes
Metopus spiculatus
Metopus vestitus
Metopid sp. 1
Metopus paravestitus
Metopus cf. paravestitus
Metopus contortus
Metopid sp. 2
Metopus major
Metopus sp.
Metopid sp. 3
Metopus parapelitus
GOOSE2B
OM801553
OM801554
MF360251
NARROWRIVER
MT825671
F26
FAMO1
KY432957
MF360250
QDUF
OP880426
LC062510
SALT15A
JUD82
BLAME
Z29516
BI27A
OM801555
METOPIDA
Metopidae
Brachonella
Brachonella contorta
Brachonella pulchra
Brachonella comma
AJ009664
AJ009665
GRANCA
JACO
LEDY (3)
VELKAL (2)
KX776468
MH108621
BOPAT (4)
OK335177
Metopus es
MALKAL (2)
VYSLU2 (4)
KUNRAMET (2)
KX776462
GDUKABAM (4)
TRIANGLE (10)
KX776464
HAC-clade
BoPl
PiCa
IAC-clade
Planometopus contractus
Bothrostoma spp.
Castula sp.OKHRE
Castula specialis
Pileometopus lynni
Castula fusca
Castula sp. 1
Castula flexibilis
Heterometopus spp.
Longitaenia gibba
Metopus boletus
Metopid sp. 7
Metopus laminarius
Metopid sp. 6
Metopus minor
Atopospira spp.
Idiometopus turbo
Metopidae SK
Parametopidium circumlabens
Metopid sp. 4
Metopid sp. 5
Metopus yantaiensis
Metopus hasei
MN604063
LERMETZ OP034700
LEDY (3)
MN604065
LERMONSTR OP034702
LERM10C OP034704
AJ009661
MN604066
DERMI
MN604069
MH086825
ALOOA1
MH086821
KF607088
LERMA5 (2)
MH086822
MG966507
MT231936
KP712777
ELJAD1
GRIDI
KY432959
CLEVELANDELLIDA
Nyctotheridae + Clevelandellidae
18S rRNA
ML 100
0.1
Marine/brackish
Freshwater
Soil
Endobiotic
Unknown
Methanoregula
Methanocorpusculum
Methanobacterium
Methanobrevibacter
NR028254 Methanofollis liminatans 4140G
NR_028856 Methanoculleus submarinus
NR028253 Methanoculleus palmolei
NR_044727 Methanoplanus limicola
KX787709 Methanomicrobium MC40
NR044726 Methanomicrobium mobil
EU721752 Methanoplanus D003024F01
NR_152660 Methanomicrobium antiquum
EU721746 Methanoplanus sp D003011I02
NR_028225 Methanogenium marinum
NR074177 Methanospirillum hungatei JF1
NR112799 Methanolinea mesophila
Methanoregula (Mr.)
NR074180 Methanoregula boonei 6A8
JX462514 Methanoregula NSArc2
Mr. sym. Caenomorpha medusula GROSERDU_NS
Mr. sym. Caenomorpha medusula GROSERDU_1
Mr. sym. Caenomorpha medusula GROSERDU_2
JF789587 Methanoregula TS1A275
Mr. sym. Longitaenia gibba DERMI_NS
LC466985 symbiont of Scuticociliatia GW7
Mr. sym. Tropidoatractus ariella KUCR13mixKUTRI_3
Mr. sym. Tropidoatractus ariella KUCR13
Mr. sym. Tropidoatractus ariella KUCR13mixKUSUS_1
Mr. sym. Tropidoatractus ariella KUCR13mixKUTRI_1
Mr. sym. Tropidoatractus ariella KUCR13mixKUTRI_2
Mr. sym. Tropidoatractus ariella KUCR13mixKUTRI_NS
Mr. sym. Tropidoatractus ariella KUCR13mixKUSUS_NS
Mr. sym. Tropidoatractus ariella KUCR13mixKUSUS_4
Mr. sym. Tropidoatractus ariella KUCR13mixKUSUS_2
Mr. sym. Tropidoatractus ariella KUCR13mixKUSUS_3
Mr. sym. Tropidoatractus sp. LERMA5_NS
Mr. sym. Tropidoatractus sp. LERMA5
Mr. sym. Tropidoatractus ariella RAJ
Mr. sym. Tropidoatractus ariella RAJ_NS
Mr. sym. Tropidoatractus levanderi MORETESmixMORLE_2
Mr. sym. Tropidoatractus levanderi MORETESmixMORLE_1
Mr. sym. Tropidoatractus levanderi MORETES_1
Mr. sym. Tropidoatractus levanderi MORETES_2
Mr. sym. Tropidoatractus levanderi MORETESmixMORLE_NS
Mr. sym. Tropidoatractus levanderi MORETESmixMORLE_3
Mr. sym. Tropidoatractus levanderi LUKIFOREST_1
Mr. sym. Tropidoatractus levanderi LUKIFOREST_2
Mr. sym. Tropidoatractus levanderi LUKIFOREST_NS
Mr. sym. Brachonella contorta JACO
Mr. sym. Brachonella contorta JACO_NS
CP121470 Methanoregula pelomyxae
Mr. sym. Tropidoatractus levanderi RIOT
Mr. sym. Tropidoatractus levanderi RIOTmixRIOTKU_NS
Mr. sym. Tropidoatractus levanderi RIOTmixRIOTKU_1
NR102441 Methanoregula formicica SMSP
Mr. sym. Tropidoatractus levanderi RIOZ
Mr. sym. Brachonella contorta GRANCA
Mr. sym. Tropidoatractus levanderi KAENG_1
Mr. sym. Tropidoatractus levanderi KAENG_2
Mr. sym. Tropidoatractus levanderi KAENG_NS
Mr. sym. Tropidoatractus levanderi VERNON_NS
Mr. sym. Tropidoatractus levanderi VERNON
Mr. sym. Brachonella contorta VELKAL
Mr. sym. Brachonella contorta VELKAL_NS
Mr. sym. of marine metopid NARROWRIVER
Mr. sym. Brachonella contorta LEDY_3
Mr. sym. Brachonella contorta LEDY_1
Mr. sym. Brachonella contorta LEDY_2
KJ955705 Methanoregula BEMA12D3C1
Mr. sym. Urostomides pullus KLAN2BC_1
Mr. sym. Urostomides pullus KLAN2BC_2
Mr. sym. Urostomides pullus KLAN2BC_NS
Mr. sym. Urostomides pullus CKAMOCBO
Mr. sym. Brachonella pulchra BOPAT_1
Mr. sym. Brachonella pulchra BOPAT_2
Mr. sym. Brachonella pulchra BOPAT_3
Mr. sym. Brachonella pulchra BOPAT_NS-1
Mr. sym. Brachonella pulchra BOPAT_NS-2
Mr. sym. Metopus es MALKAL
Mr. sym. Metopus es MALKAL_NS
16S rRNA
0.05

Figure S3. Maximum likelihood phylogenetic trees based on 18S (left) and 16S (right) rRNA gene sequences showing the connection between ciliate hosts and all obtained symbiotic Sanger sequences belonging to the genus *Methanoregula* (in bold). The habitat of the ciliate is depicted in the 18S tree. The scale bar represents 5 substitutions per 100 positions. Bootstrap values below 50 are not shown.

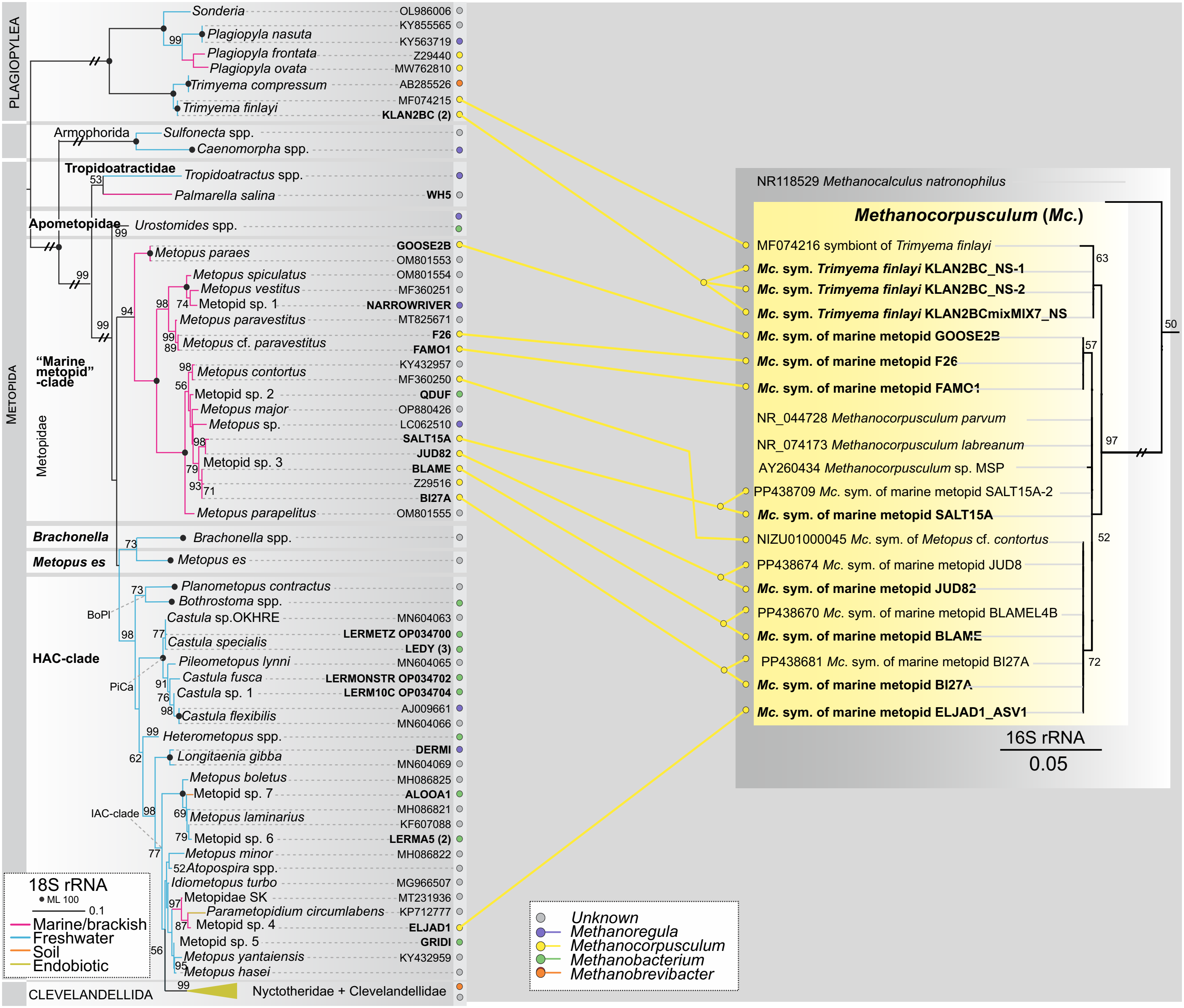

PLAGIOPYLEA
Sonderia OL986006
Plagiopyla nasuta KY855565
KY563719
Plagiopyla frontata Z29440
Plagiopyla ovata MW762810
Trimyema compressum AB285526
Trimyema finlayi MF074215
KLAN2BC (2)
Armophorida
Sulfonecta spp.
Caenomorpha spp.
Tropidoatractidae
Tropidoatractus spp.
Palmarella salina WH5
Apometopidae
Urostomides spp.
Metopus paraes GOOSE2B
OM801553
Metopus spiculatus OM801554
Metopus vestitus MF360251
Metopid sp. 1 NARROWRIVER
Metopus paravestitus MT825671
F26
Metopus cf. paravestitus FAMO1
Metopus contortus KY432957
MF360250
Metopid sp. 2 QDUF
Metopus major OP880426
Metopus sp. LC062510
SALT15A
JUD82
Metopid sp. 3 BLAME
Z29516
BI27A
Metopus parapelitus OM801555
"Marine metopid" -clade
Metopidae
METOPIDA
Brachonella
Brachonella spp.
Metopus es
Metopus es
Planometopus contractus
Bothrostoma spp.
BoPl
Castula sp.OKHRE MN604063
Castula specialis LERMETZ OP034700
LEDY (3)
HAC-clade
Pileometopus lynni MN604065
PiCa
Castula fusca LERMONSTR OP034702
Castula sp. 1 LERM10C OP034704
Castula flexibilis AJ009661
MN604066
Heterometopus spp.
Longitaenia gibba DERMI
MN604069
Metopus boletus MH086825
Metopid sp. 7 ALOOA1
IAC-clade
MH086821
Metopus laminarius KF607088
Metopid sp. 6 LERMA5 (2)
Metopus minor MH086822
Atopospira spp.
Idiometopus turbo MG966507
Metopidae SK MT231936
Parametopidium circumlabens KP712777
Metopid sp. 4 ELJAD1
Metopid sp. 5 GRIDI
Metopus yantaiensis KY432959
Metopus hasei
CLEVELANDELLIDA
Nyctotheridae + Clevelandellidae
18S rRNA
ML 100
0.1
Marine/brackish
Freshwater
Soil
Endobiotic
Unknown
Methanoregula
Methanocorpusculum
Methanobacterium
Methanobrevibacter
NR118529 Methanocalculus natronophilus
Methanocorpusculum (Mc.)
MF074216 symbiont of Trimyema finlayi
Mc. sym. Trimyema finlayi KLAN2BC_NS-1
Mc. sym. Trimyema finlayi KLAN2BC_NS-2
Mc. sym. Trimyema finlayi KLAN2BCmixMIX7_NS
Mc. sym. of marine metopid GOOSE2B
Mc. sym. of marine metopid F26
Mc. sym. of marine metopid FAMO1
NR_044728 Methanocorpusculum parvum
NR_074173 Methanocorpusculum labreanum
AY260434 Methanocorpusculum sp. MSP
PP438709 Mc. sym. of marine metopid SALT15A-2
Mc. sym. of marine metopid SALT15A
NIZU01000045 Mc. sym. of Metopus cf. contortus
PP438674 Mc. sym. of marine metopid JUD8
Mc. sym. of marine metopid JUD82
PP438670 Mc. sym. of marine metopid BLAMEL4B
Mc. sym. of marine metopid BLAME
PP438681 Mc. sym. of marine metopid BI27A
Mc. sym. of marine metopid BI27A
Mc. sym. of marine metopid ELJAD1_ASV1
16S rRNA
0.05

Figure S4. Maximum likelihood phylogenetic trees based on 18S (left) and 16S (right) rRNA gene sequences showing the connection between ciliate hosts and all obtained symbiotic Sanger sequences belonging to the genus *Methanocorpusculum* (in bold). The habitat of the ciliate is depicted in the 18S tree. The scale bar represents 5 substitutions per 100 positions. Bootstrap values below 50 are not shown.

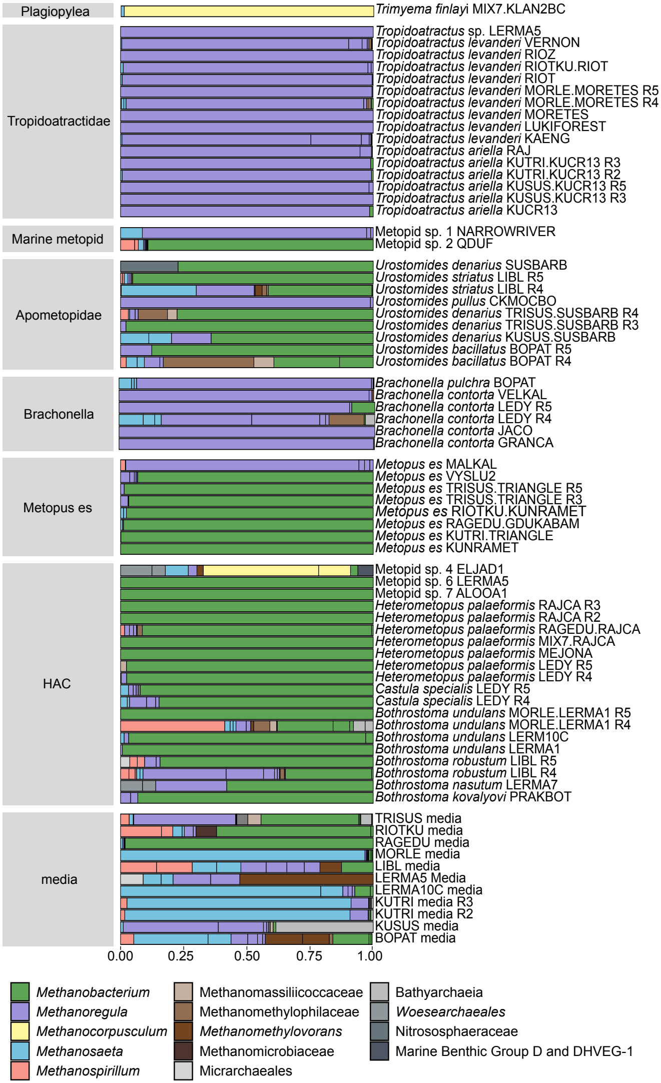

Plagiopylea
Trimyema finlayi MIX7.KLAN2BC
Tropidoatractidae
Tropidoatractus sp. LERMA5
Tropidoatractus levanderi VERNON
Tropidoatractus levanderi RIOZ
Tropidoatractus levanderi RIOTKU.RIOT
Tropidoatractus levanderi RIOT
Tropidoatractus levanderi MORLE.MORETES R5
Tropidoatractus levanderi MORLE.MORETES R4
Tropidoatractus levanderi MORETES
Tropidoatractus levanderi LUKIFOREST
Tropidoatractus levanderi KAENG
Tropidoatractus ariella RAJ
Tropidoatractus ariella KUTRI.KUCR13 R3
Tropidoatractus ariella KUTRI.KUCR13 R2
Tropidoatractus ariella KUSUS.KUCR13 R5
Tropidoatractus ariella KUSUS.KUCR13 R3
Tropidoatractus ariella KUCR13
Marine metopid
Metopid sp. 1 NARROWRIVER
Metopid sp. 2 QDUF
Apometopidae
Urostomides denarius SUSBARB
Urostomides striatus LIBL R5
Urostomides striatus LIBL R4
Urostomides pullus CKMOCBO
Urostomides denarius TRISUS.SUSBARB R4
Urostomides denarius TRISUS.SUSBARB R3
Urostomides denarius KUSUS.SUSBARB
Urostomides bacillatus BOPAT R5
Urostomides bacillatus BOPAT R4
Brachonella
Brachonella pulchra BOPAT
Brachonella contorta VELKAL
Brachonella contorta LEDY R5
Brachonella contorta LEDY R4
Brachonella contorta JACO
Brachonella contorta GRANCA
Metopus es
Metopus es MALKAL
Metopus es VYSLU2
Metopus es TRISUS.TRIANGLE R5
Metopus es TRISUS.TRIANGLE R3
Metopus es RIOTKU.KUNRAMET
Metopus es RAGEDU.GDUKABAM
Metopus es KUTRI.TRIANGLE
Metopus es KUNRAMET
HAC
Metopid sp. 4 ELJAD1
Metopid sp. 6 LERMA5
Metopid sp. 7 ALOOA1
Heterometopus palaeformis RAJCA R3
Heterometopus palaeformis RAJCA R2
Heterometopus palaeformis RAGEDU.RAJCA
Heterometopus palaeformis MIX7.RAJCA
Heterometopus palaeformis MEJONA
Heterometopus palaeformis LEDY R5
Heterometopus palaeformis LEDY R4
Castula specialis LEDY R5
Castula specialis LEDY R4
Bothrostoma undulans MORLE.LERMA1 R5
Bothrostoma undulans MORLE.LERMA1 R4
Bothrostoma undulans LERM10C
Bothrostoma undulans LERMA1
Bothrostoma robustum LIBL R5
Bothrostoma robustum LIBL R4
Bothrostoma nasutum LERMA7
Bothrostoma kovalyovi PRAKBOT
media
TRISUS media
RIOTKU media
RAGEDU media
MORLE media
LIBL media
LERMA5 Media
LERMA10C media
KUTRI media R3
KUTRI media R2
KUSUS media
BOPAT media
0.00
0.25
0.50
0.75
1.00
Methanobacterium
Methanoregula
Methanocorpusculum
Methanosaeta
Methanospirillum
Methanomassiliicoccaceae
Methanomethylophilaceae
Methanomethylovorans
Methanomicrobiaceae
Micrarchaeales
Bathyarchaeia
Woesearchaeales
Nitrososphaeraceae
Marine Benthic Group D and DHVEG-1

Figure S5. Barplot showing the relative abundances of the archaeal genera recovered from 16S rRNA gene amplicon sequencing from each ciliate sample and from the media. Samples are grouped according to the host clade.

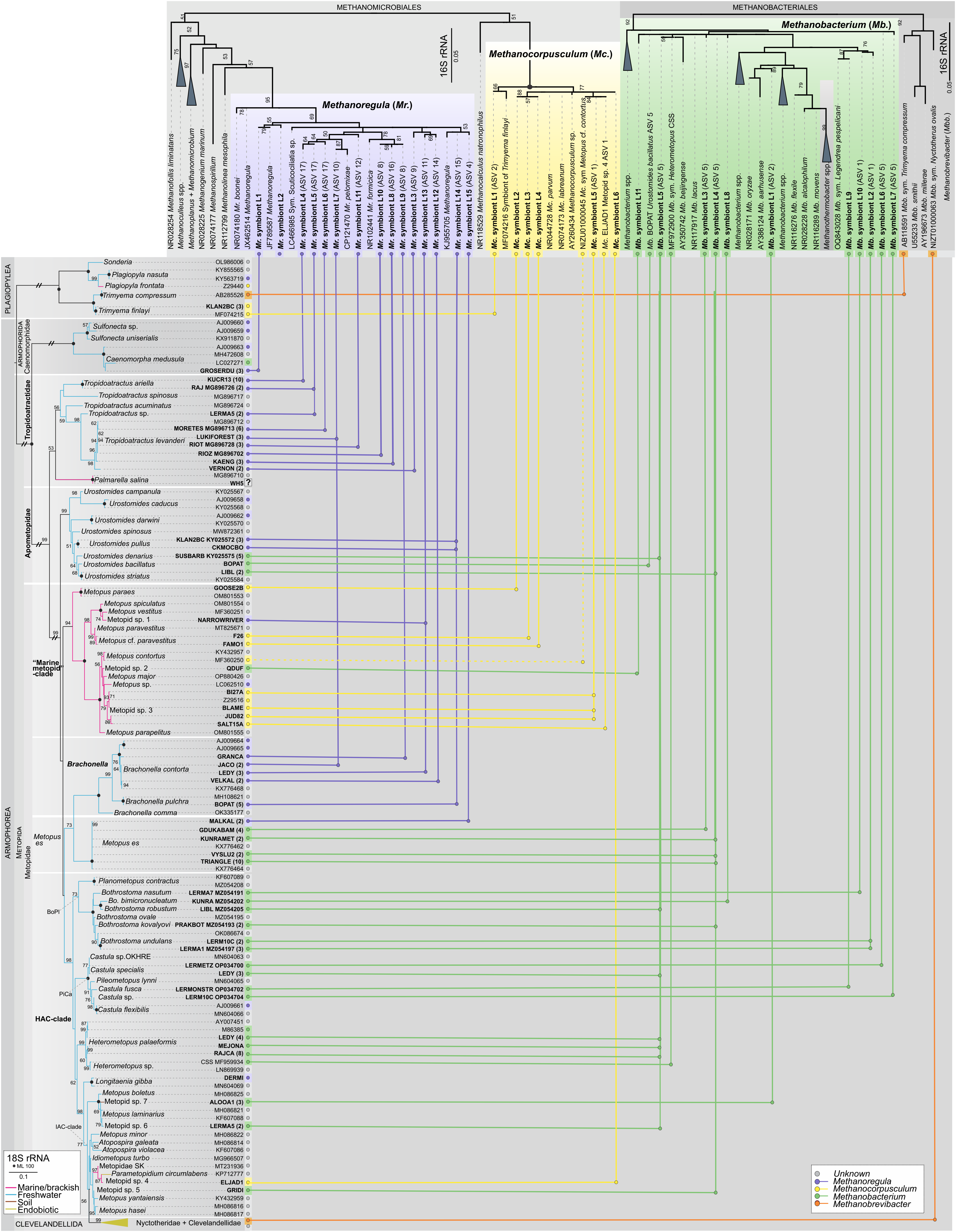

METHANOMICROBIALES
METHANOBACTERIALES
Methanoregula (Mr.)
Methanocorpusculum (Mc.)
Methanobacterium (Mb.)
16S rRNA
0.05
NR028254 Methanofollis liminatans
Methanoculleus spp.
Methanoplanus + Methanomicrobium
NR028225 Methanogenium marinum
NR074177 Methanospirillum
NR112799 Methanolinea mesophila
NR074180 Mr. boonei
JX462514 Methanoregula
Mr. symbiont L1
JF789587 Methanoregula
Mr. symbiont L2
LC466985 Sym. Scuticociliatia sp.
Mr. symbiont L4 (ASV 17)
Mr. symbiont L5 (ASV 17)
Mr. symbiont L6 (ASV 17)
Mr. symbiont L7 (ASV 10)
CP121470 Mr. pelomixae
Mr. symbiont L11 (ASV 12)
NR102441 Mr. formicica
Mr. symbiont L10 (ASV 8)
Mr. symbiont L8 (ASV 16)
Mr. symbiont L9 (ASV 8)
Mr. symbiont L3 (ASV 9)
Mr. symbiont L13 (ASV 11)
Mr. symbiont L12 (ASV 14)
KJ955705 Methanoregula
Mr. symbiont L14 (ASV 15)
Mr. symbiont L15 (ASV 4)
NR118529 Methanocalculus natronophilus
Mc. symbiont L1 (ASV 2)
MF074216 Symbiont of Trimyema finlayi
Mc. symbiont L2
Mc. symbiont L3
Mc. symbiont L4
NR044728 Mc. parvum
NR074173 Mc. labreanum
AY260434 Methanocorpusculum sp.
NIZU01000045 Mc. sym Metopus cf. contortus
Mc. symbiont L5 (ASV 1)
Mc. ELJAD1 Metopid sp. 4 ASV 1
Mc. symbiont L6
Methanobacterium spp.
Mb. symbiont L11
Mb. BOPAT Urostomides bacillatus ASV 5
Mb. symbiont L5 (ASV 5)
MF972900 Mb. sym. Heterometopus CSS
AY350742 Mb. beijingense
NR117917 Mb. lacus
Mb. symbiont L3 (ASV 5)
Mb. symbiont L4 (ASV 5)
Mb. symbiont L8
Methanobacterium spp.
NR028171 Mb. oryzae
AY386124 Mb. aarhusense
Mb. symbiont L1 (ASV 2)
Methanobacterium spp.
NR116276 Mb. flexile
NR028228 Mb. alcaliphilum
NR116289 Mb. movens
Methanothermobacter spp.
OQ843028 Mb. sym. Legendrea pespelicani
Mb. symbiont L9
Mb. symbiont L10 (ASV 1)
Mb. symbiont L2 (ASV 1)
Mb. symbiont L6 (ASV 5)
Mb. symbiont L7 (ASV 5)
AB118591 Mbb. sym. Trimyema compressum
U55233 Mbb. smithii
AY196673 Mbb. millerae
NIZT01000063 Mbb. sym. Nyctotherus ovalis
Methanobrevibacter (Mbb.)
PLAGIOPYLEA
ARMOPHORIDA Caenomorphidae
Tropidoatractidae
Apometopidae
"Marine metopid"-clade
ARMOPHOREA
METOPIDA
Metopidae
Brachonella
Metopus es
BoPl
PiCa
HAC-clade
IAC-clade
CLEVELANDELLIDA
Sonderia
Plagiopyla nasuta
Plagiopyla frontata
Trimyema compressum
Trimyema finlayi
Sulfonecta sp.
Sulfonecta uniserialis
Caenomorpha medusula
Tropidoatractus ariella
Tropidoatractus spinosus
Tropidoatractus acuminatus
Tropidoatractus sp.
Tropidoatractus levanderi
Palmarella salina
Urostomides campanula
Urostomides caducus
Urostomides darwini
Urostomides spinosus
Urostomides pullus
Urostomides denarius
Urostomides bacillatus
Urostomides striatus
Metopus paraes
Metopus spiculatus
Metopus vestitus
Metopid sp. 1
Metopus paravestitus
Metopus cf. paravestitus
Metopus contortus
Metopid sp. 2
Metopus major
Metopus sp.
Metopid sp. 3
Metopus parapelitus
Brachonella contorta
Brachonella pulchra
Brachonella comma
Metopus es
Planometopus contractus
Bothrostoma nasutum
Bo. bimicronucleatum
Bothrostoma robustum
Bothrostoma ovale
Bothrostoma kovalyovi
Bothrostoma undulans
Castula sp.OKHRE
Castula specialis
Pileometopus lynni
Castula fusca
Castula sp.
Castula flexibilis
Heterometopus palaeformis
Heterometopus sp.
Longitaenia gibba
Metopus boletus
Metopid sp. 7
Metopus laminarius
Metopid sp. 6
Metopus minor
Atopospira galeata
Atopospira violacea
Idiometopus turbo
Metopidae SK
Parametopidium circumlabens
Metopid sp. 4
Metopid sp. 5
Metopus yantaiensis
Metopus hasei
Nyctotheridae + Clevelandellidae
OL986006
KY855565
KY563719
Z29440
AB285526
KLAN2BC (3)
MF074215
AJ009660
AJ009659
KX911870
AJ009663
MH472608
LC027271
GROSERDU (3)
KUCR13 (10)
RAJ MG896726 (2)
MG896717
MG896724
LERMA5 (2)
MG896712
MORETES MG896713 (6)
LUKIFOREST (3)
RIOT MG896728 (3)
RIOZ MG896702
KAENG (3)
VERNON (2)
MG896710
WH5
KY025567
AJ009658
KY025568
AJ009662
KY025570
MW872361
KLAN2BC KY025572 (3)
CKMOCBO
SUSBARB KY025575 (5)
BOPAT
LIBL (2)
KY025584
GOOSE2B
OM801553
OM801554
MF360251
NARROWRIVER
MT825671
F26
FAMO1
KY432957
MF360250
QDUF
OP880426
LC062510
BI27A
Z29516
BLAME
JUD82
SALT15A
OM801555
AJ009664
AJ009665
GRANCA
JACO (2)
LEDY (3)
VELKAL (2)
KX776468
MH108621
BOPAT (5)
OK335177
MALKAL (2)
GDUKABAM (4)
KUNRAMET (2)
KX776462
VYSLU2 (2)
TRIANGLE (10)
KX776464
KF607089
MZ054208
LERMA7 MZ054191
KUNRA MZ054202
LIBL MZ054205
MZ054195
PRAKBOT MZ054193 (2)
OK086674
LERM10C (2)
LERMA1 MZ054197 (3)
MN604063
LERMETZ OP034700
LEDY (3)
MN604065
LERMONSTR OP034702
LERM10C OP034704
AJ009661
MN604066
AY007451
M86385
LEDY (4)
MEJONA
RAJCA (8)
CSS MF959934
LN869939
DERMI
MN604069
MH086825
ALOOA1 (3)
MH086821
KF607088
LERMA5 (2)
MH086822
MH086814
KF607086
MG966507
MT231936
KP712777
ELJAD1
GRIDI
KY432959
MH086816
MH086817
18S rRNA
ML 100
0.1
Marine/brackish
Freshwater
Soil
Endobiotic
Unknown
Methanoregula
Methanocorpusculum
Methanobacterium
Methanobrevibacter

Figure S6. Maximum likelihood phylogenetic trees based on 18S (left) and 16S (right) rRNA gene sequences showing the connection between ciliate hosts and their respective methanogenic symbionts. The habitat of the ciliate is depicted in the 18S tree. The number of 16S rRNA Sanger sequences obtained per ciliate strain is in brackets. The corresponding dominant ASVs which are 100% identical to the symbiont lineages are indicated in the 16S tree in brackets. Bootstrap values below 50 are not shown. The scale bar represents 10 substitutions per 100 positions in the 18S tree and 5 substitutions per 100 positions in the 16S trees.

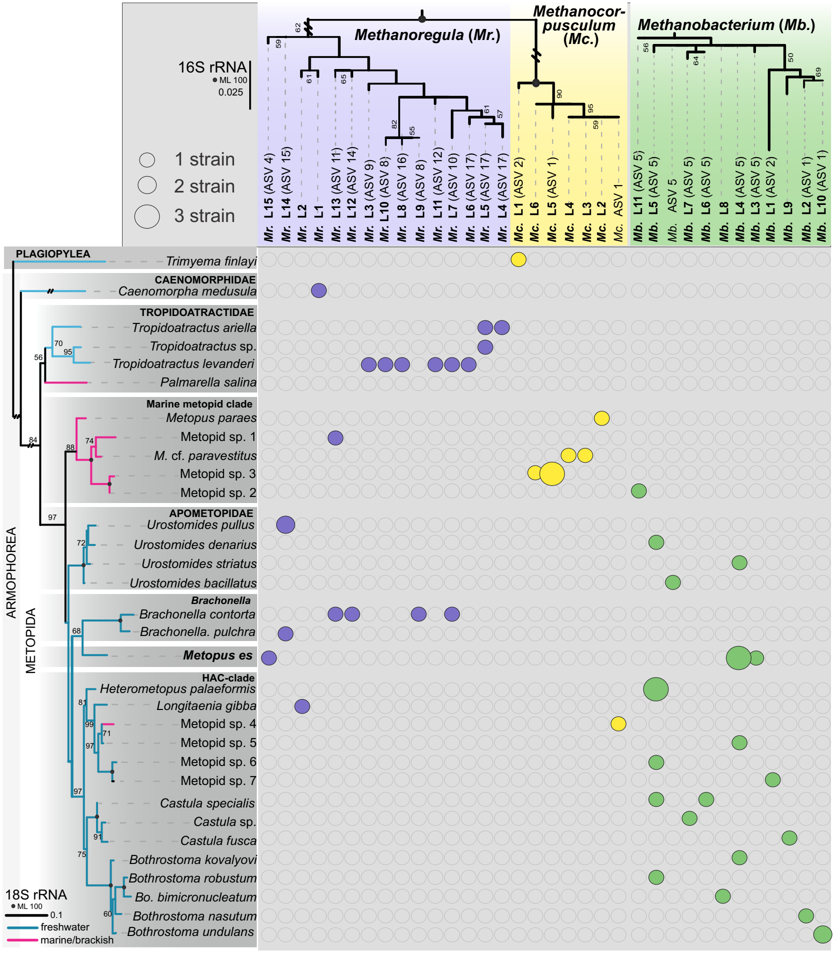
Methanoregula (Mr.)
Methanocorpusculum (Mc.)
Methanobacterium (Mb.)
16S rRNA
ML 100
0.025
1 strain
2 strain
3 strain
Mr. L15 (ASV 4)
Mr. L14 (ASV 15)
Mr. L2
Mr. L1
Mr. L13 (ASV 11)
Mr. L12 (ASV 14)
Mr. L3 (ASV 9)
Mr. L10 (ASV 8)
Mr. L8 (ASV 16)
Mr. L9 (ASV 8)
Mr. L11 (ASV 12)
Mr. L7 (ASV 10)
Mr. L6 (ASV 17)
Mr. L5 (ASV 17)
Mr. L4 (ASV 17)
Mc. L1 (ASV 2)
Mc. L6
Mc. L5 (ASV 1)
Mc. L4
Mc. L3
Mc. L2
Mc. ASV 1
Mb. L11 (ASV 5)
Mb. L5 (ASV 5)
Mb. ASV 5
Mb. L7 (ASV 5)
Mb. L6 (ASV 5)
Mb. L8
Mb. L4 (ASV 5)
Mb. L3 (ASV 5)
Mb. L1 (ASV 2)
Mb. L9
Mb. L2 (ASV 1)
Mb. L10 (ASV 1)
PLAGIOPYLEA
Trimyema finlayi
CAENOMORPHIDAE
Caenomorpha medusula
TROPIDOATRACTIDAE
Tropidoatractus ariella
Tropidoatractus sp.
Tropidoatractus levanderi
Palmarella salina
Marine metopid clade
Metopus paraes
Metopid sp. 1
M. cf. paravestitus
Metopid sp. 3
Metopid sp. 2
APOMETOPIDAE
Urostomides pullus
Urostomides denarius
Urostomides striatus
Urostomides bacillatus
Brachonella
Brachonella contorta
Brachonella. pulchra
Metopus es
HAC-clade
Heterometopus palaeformis
Longitaenia gibba
Metopid sp. 4
Metopid sp. 5
Metopid sp. 6
Metopid sp. 7
Castula specialis
Castula sp.
Castula fusca
Bothrostoma kovalyovi
Bothrostoma robustum
Bo. bimicronucleatum
Bothrostoma nasutum
Bothrostoma undulans
ARMOPHOREA
METOPIDA
18S rRNA
ML 100
0.1
freshwater
marine/brackish

Figure S7. Distribution of the methanogenic symbiont lineages (top tree) across the host ciliate species (left tree). The number of host strains per each ciliate species is represented by bubbles.

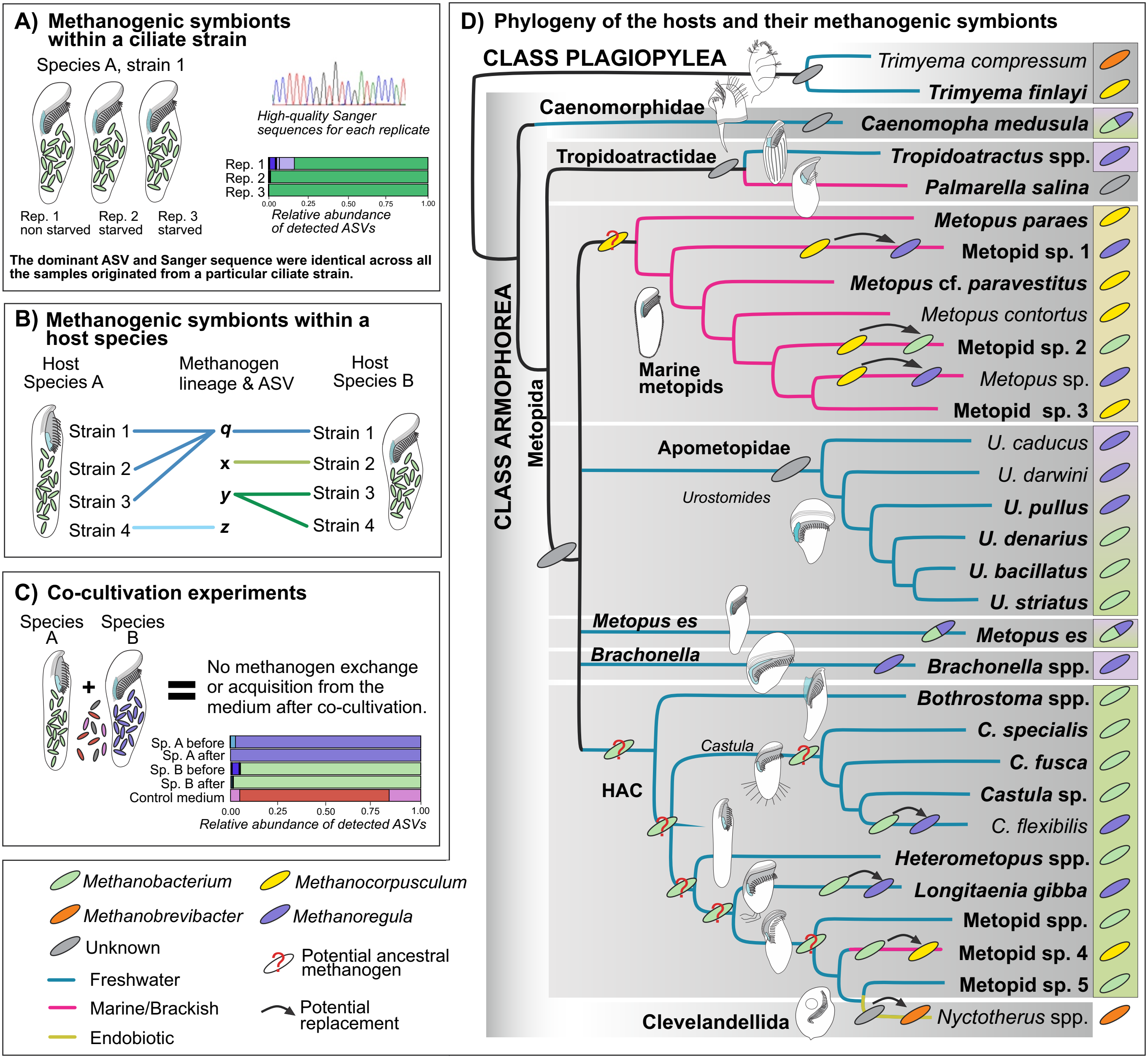
A) Methanogenic symbionts within a ciliate strain
Species A, strain 1
High-quality Sanger sequences for each replicate
Rep. 1
Rep. 2
Rep. 3
0.00 0.25 0.50 0.75 1.00
Relative abundance of detected ASVs
Rep. 1 non starved
Rep. 2 starved
Rep. 3 starved
The dominant ASV and Sanger sequence were identical across all the samples originated from a particular ciliate strain.
B) Methanogenic symbionts within a host species
Host Species A
Methanogen lineage & ASV
Host Species B
Strain 1
Strain 2
Strain 3
Strain 4
q
x
y
z
Strain 1
Strain 2
Strain 3
Strain 4
C) Co-cultivation experiments
Species A
Species B
No methanogen exchange or acquisition from the medium after co-cultivation.
Sp. A before
Sp. A after
Sp. B before
Sp. B after
Control medium
0.00 0.25 0.50 0.75 1.00
Relative abundance of detected ASVs
Methanobacterium
Methanocorpusculum
Methanobrevibacter
Methanoregula
Unknown
Potential ancestral methanogen
Freshwater
Marine/Brackish
Potential replacement
Endobiotic
D) Phylogeny of the hosts and their methanogenic symbionts
CLASS PLAGIOPYLEA
Trimyema compressum
Trimyema finlayi
Caenomorphidae
Caenomopha medusula
Tropidoatractidae
Tropidoatractus spp.
Palmarella salina
Metopus paraes
Metopid sp. 1
Metopus cf. paravestitus
Metopus contortus
Metopid sp. 2
Metopus sp.
Metopid sp. 3
Marine metopids
CLASS ARMOPHOREA
Metopida
Apometopidae
Urostomides
U. caducus
U. darwini
U. pullus
U. denarius
U. bacillatus
U. striatus
Metopus es
Metopus es
Brachonella
Brachonella spp.
Bothrostoma spp.
C. specialis
C. fusca
Castula
Castula sp.
C. flexibilis
HAC
Heterometopus spp.
Longitaenia gibba
Metopid spp.
Metopid sp. 4
Metopid sp. 5
Clevelandellida
Nyctotherus spp.

Figure S8A-D. Summary of results. A, Methanogenic symbionts within a strain are genetically identical across replicates regardless of starved or non-starved conditions. B, Methanogenic symbionts of different strains within a ciliate species (intraspecific) are genetically divergent in most cases. C, Methanogenic symbionts of co-occurring unrelated ciliate species are maintained rather than exchanged, and the archeal communities from the control medium differ from those in the ciliates. D, Schematic phylogenetic tree of the ciliate hosts and their respective methanogenic symbionts. Potential ancestral and replacements of the methanogenic symbionts are shown. Species examined in this study are in bold.
