# Supplementary material for: Methanogenic symbionts of anaerobic ciliates are host and habitat specific
Source: ISME J. 2024 Aug 20;18(1):wrae164. doi: 10.1093/ismejo/wrae164 (PMC11378729; doi:10.1093/ismejo/wrae164)
Supplement: Supplementary_material [file supplementary_material.zip › Supplemental_material_wrae164.docx]

**Methanogenic symbionts of anaerobic ciliates are host- and habitat-specific**

Daniel Méndez-Sánchez^1, a, *^, Anna Schrecengost^2, a^, Johana Rotterová^1, 2, b^, Kateřina Koštířová^1^, Roxanne A. Beinart^2^, Ivan Čepička^1, *^

^1^Department of Zoology, Faculty of Science, Charles University, Viničná 7, 128 00 Prague 2, Czech Republic.

^2^Graduate School of Oceanography, University of Rhode Island, Narragansett, Rhode Island, USA.

^a^Both authors contributed equally to the work.

^b^Current address: Department of Marine Sciences, University of Puerto Rico Mayagüez, Mayagüez, Puerto Rico, USA.

^*^Corresponding authors: Daniel Méndez-Sánchez, Department of Zoology, Faculty of Science, Charles University, Viničná 7, Prague, 128 00 Prague 2, Czech Republic, email: [mendezsd@natur.cuni.cz](mailto:mendezsd@natur.cuni.cz), and Ivan Čepička, Department of Zoology, Faculty of Science, Charles University, Viničná 7, Prague, 128 00 Prague 2, Czech Republic, tel: (+420) 221 951 812, email: [ivan.cepicka@natur.cuni.cz](mailto:ivan.cepicka@natur.cuni.cz)

**Supplemental material**

1. **Supplemental methods**
2. **Supplemental tables**
3. **Supplemental files**
4. **Supplemental methods**

**Origin and cultivation of anaerobic ciliates**

Sediment samples from soil, freshwater, and brackish/marine aquatic environments were collected using 15- or 50-ml Falcon tubes of or small jars from different geographical regions within the period of 2010 and 2021 (Table S1). 1- to 2-ml of the collected sediment was inoculated in 15-ml falcon tubes containing 10-ml of Sonneborn’s *Paramecium* medium: ATCC medium #802 and/or #1525 for freshwater and brackish/marine samples, respectively. Tubes were tightly closed to avoid oxygen exchange, and only open during sub-culturing and examination. Cultures were examined under optical microscope. If ciliates were present, then the sediment was transferred into fresh media every week. After several weeks or months of sub-culturing, the ciliate species became stable and abundant enough for culture maintenance. Sub-culturing was done biweekly in the respective medium. All the examined cultures were polyxenic, that means that one or two ciliate species and several unknown prokaryotic species were present in the culture (Rotterová and Čepička 2019). Cultures were kept in darkness at room temperature (23 °C) for up to 10 years until their examination.

A total of 54 ciliate strains distributed in 47 cultures were used in this study, one culture had three ciliate species, five had two species each, and the remaining forty-one had a single ciliate species. Forty-two strains are from freshwater origin, 11 from marine/brackish, and one from soil. A total of 32 ciliate species were identified based on 18S rRNA gene sequences from individual washed cells: 30 metopids (Armophorea, Metopida), one *Caenomorpha* (Armophorea, Armophorida), and one *Trimyema* (Plagiopylea, Plagiopylida) (Table S1).

**Fluorescence in situ hybridization**

Cells of selected strains were fixed in 4% paraformaldehyde for 30 min at room temperature, filtered onto 0.22 µm Isopore filters (Millipore), and washed with 1x phosphate buffered saline. Filter sections were embedded in 0.2% MetaPhor agarose (Lonza). Cells were permeabilized using lysozyme (10 mg/ml) for 1 hour at 37 °C. An additional permeabilization step in 0.5% Triton X-100 for 30 minutes was added for some strains if needed. Hybridization was carried out at 46 °C for 2 hours with the following formamide concentrations: 40% for ARC915, 20% for MG1200b (Raskin et al. 1994; Crocetti et al. 2006). Filters were then washed in buffer (20 mM Tris-HCl, pH 8.0; 5 mM EDTA, pH 8.0; 70 mM NaCl; 0.01% SDS) at 48 °C for 15 minutes, then quickly washed in cold deionized water, and finally dipped in cold 96% ethanol. Air-dried filter sections were mounted on glass slides using Vectashield with DAPI (Vector Laboratories). Preparations were observed using a confocal microscope Leica TCS SP8 (equipment of Laboratory of Confocal and Fluorescence Microscopy, Faculty of Science, Charles University). Image deconvolution was performed using SVI Huygens Software (version 24.04) with standard settings. Subsequent image processing was done in FIJI software (version 2.15.1). Projections of multiple focal Z-planes were stacked using the Z-stack function in FIJI software.

**Transmission electron microscopy**

Cells were centrifuged and fixed in cacodylate buffer with 2.5% glutaraldehyde (Polysciences), then washed three times in 0.1M cacodylate buffer and postfixed with 2% OsO_4_ in 0.1M cacodylate buffer, subsequently washed, dehydrated in ethanol series and graduate ethanol-acetone, finally the pellet was embedded in resin and polymerized. Blocks were sectioned using an ultramicrotome and examined under a JEOL JEM-1011 transmission electron microscope.

**Cell picking and sample preparation**

To avoid potential contaminants, right before the sample preparation, glass micropipettes, depression slides with several wells, 1.5-ml Eppendorf tubes, 0.22 µm pore-size filters, syringes with fresh medium or distilled water were UV-treated and/or cleaned with DNA AWAY Surface Decontaminant (Thermo Scientific). For each ciliate strain, one or two drops from the culture’s sediment was added into one of the depression slide wells. Five to 10 cells (Table S2) were handpicked with glass micropipettes under an inverted microscope and transferred 5 or 6 times into a new well with culture medium or distilled water to remove potential free-living bacteria (washing). Washed cells were placed in a 1.5-ml microcentrifuge tube containing 0.22 µm of sterile Sonneborn’s *Paramecium* medium or distilled water to induce starvation. After a time of starvation, ranging from 0.5 to 24 hours (Table S2), cells were observed under a phase contrast microscope to detect the presence of food vacuoles. The absence of food vacuoles is an indication of starvation (Omar et al. 2017; Beinart et al. 2018; Beinart and Rotterová 2019). Starved cells were transferred five times with water or sterile culture medium and lastly harvested and stored in 30-µl of UV-treated DNA/RNA shield (Zymo Research, Irvine, CA, USA) (Beinart and Rotterová 2019). Starved cells were also observed under fluorescence microscopy as described above. In a few cases, starvation altered the cell conditions, e.g., leading to globular cells or encystation, resulting in not-well-starved cells, but cells were stored in DNA/RNA Shield regardless of condition. Also, non-starved but washed cells from most of the strains were added directly to the UV-treated DNA/RNA Shield. Additionally, during the sample preparation, ciliates were removed from the initial drops of the culture’s sediment using glass micropipettes to obtain about 30-µl of culture medium containing the free-living prokaryotic community (control medium), which was dispensed into a tube with UV-treated DNA/RNA shield. Glass micropipettes were replaced constantly to avoid contamination. A total of 52 samples from the medium were processed (Table S11).

**FISH oligonucleotide probes**

FISH experiments were performed using cyanine 3 labelled oligonucleotide probes:ARC915 (5’-GTGCTCCCCCGCCAATTCCT-3‘) specific to all Archaea and

MG1200b (5’-CRGATAATTCGGGGCATGCTG-3‘) specific to the order Methanomicrobiales (Raskin et al, 1994; Crocetti et al., 2006). Negative controls were performed using the nonsense probe NON-EUB338 (5’-ACTCCTACGGGAGGCAGC-3’) (Wallner et al., 1993).

**PCR amplification and sequencing**

The 18S rRNA gene for each ciliate strain sample was amplified using the primers EukA (5′-AACCTGGTTGATCCTGCCAGT-3′) and EukB (5′-TGATCCATCTGCAGGTTCACCT-3′) (Medlin et al. 1988) and a DNA polymerase (PPP Master Mix, Top-Bio) with the following thermocycler parameters: 94 °C for 5 min, followed by 35 cycles of 30 s at 94 °C, 30 s at 55 °C, and 2 min at 72 °C, and final extension cycle at 72 °C for 10 min. The 16S rRNA gene fragment, approx. 450 bp (118 symbiont sequences), of methanogenic Archaea was amplified using the primers Arc915F (5′-AGGAATTGGCGGGGGAGCAC-3′) and ArcR1326 (5′-TGTGTGCAAGGAGCAGGGAC-3′) (Irbis and Ushida 2004; van Hoek et al 2000) with the same master mixed reaction thermocycler parameters, except for an annealing temperature of 60 °C. Thirteen sequences were amplified using the primers Met83F (5’-ACKGCTCAGTAACAC-3’) and Met1340R (5’-CGGTGTGTGCAAGGAG-3’) with the following thermocycler parameters: 94 °C for 1 min, followed by 30 cycles of 40 s at 94 °C, 1 min at 55 °C, and 90 s at 72 °C, and final extension cycle at 72 °C for 5 min.

Amplified DNA fragments were purified either using the Gel/PCR DNA Fragments Extraction Kit (Qiagen, Hilden, Germany), or EXOSAP (Applied Biosystems). The 18S rRNA fragments were sequenced with internal primers 577F (5́-GCCAGCACCCGCGGT-3́), 577R (5́-ACCGCGGGTGCTGGC-3́), 1055F (5′-CGGCCATGCACCACC-3′), and 1055R (5́-GGTGGTGCATGGCCG-3́) (Elwood et al. 1985); whereas the 16S rRNA fragments with primers same as for PCR plus an internal primer Met1927F (5′-GTCAGGCAACGAGCGAGACC-3′) (Narihiro and Sekiguchi 2011).

In parallel and using the same isolated DNA from each sample, amplicons for Illumina sequencing were prepared using archaea-specific barcoded primers Arch915F and ArcR1326. To obtain enough DNA and reduce PCR bias, PCR was done in triplicate for each sample. PCR products were subsequently pooled and purified using the Gel/PCR DNA Fragments Extraction Kit (Qiagen, Hilden, Germany) prior to sequencing. DNA concentration was measured on Qubit 4 Fluorometer (Thermo Fisher Scientific).

Sanger sequencing was done at the Laboratory of DNA Sequencing (Charles University, Prague, Czech Republic) on an ABI PRISM 3100 sequencer (Applied Biosystems) and at the Rhode Island Genomics and Sequencing Center (Kingston, RI USA) on a 3130xl genetic analyzer (Applied Biosystems). Sanger sequences were analyzed and assembled in Geneious Prime 11.0.9+11. Illumina sequencing (MiSeq 2x250bp) was performed at the Institute of Microbiology of the Czech Academy of Sciences, Czech Republic. A total of 140 samples (54 strains among 32 species) were sequenced, 54 (35 strains, among 20 species) of them were parallelly sequenced through Sanger and Illumina. In total 131 samples were sequenced through Sanger and 63 through Illumina. A total of 51 samples from the media (28 natural and seven artificial cultures) were analyzed, generating 55 Sanger sequences (not shown) and 11 amplicons (Table S11).

**Phylogenetic analysis of host 18S and symbiont 16S rRNA gene sequences**

A dataset containing 18S rRNA gene sequences of the 54 ciliate strains used in this study was created. We built a second dataset containing the 54 sequences plus available GenBank sequences of Armophorea as well as representatives of the class Plagiopylea (114 sequences in total) in order to cover the most updated phylogeny of Armophorea and Plagiopylea. The datasets were aligned using MAFFT (G-INS-i algorithm) (Katoh et al 2002). The alignments were manually trimmed to the primer regions, using AliView v1.28 (Larson 2014).

The 131 16S rRNA sequences of methanogenic symbionts obtained from Sanger sequencing were de-replicated with vsearch (--derep_fulllength). A total of 32 unique sequences were identified and aligned along with reference and outgroup sequences obtained from GenBank, using the SILVA SINA aligner 1.2.11 (Pruesse et al. 2012) and manually trimmed to the Arc915F/ArcR1326 primer region using AliView v1.28. In parallel, all the obtained 16S rRNA gene sequences of *Methanobacterium* (65), *Methanoregula* (56), and *Methanocorpusculum* (10) were separately aligned with the reference and outgroup sequences as above. Pairwise distance matrices (Tables S8 and S9) were computed using Clustal Omega (Larsson 2014) in Geneious Prime 11.0.9+11.

The 16S and 18S rRNA gene phylogenetic trees were generated using the Maximum likelihood method in RAxML 8.2.12 under the GTRGAMMAI model with 1000 bootstrap pseudoreplicates (Stamatakis 2014) in the web server The CIPRES Science Gateway V. 3.3 <https://www.phylo.org>.

**References**

Rotterová J, Čepička I. Cultivation protocol for anaerobic ciliates. 2019. Protocols.io <https://dx.doi.org/10.17504/protocols.io.85why7e>

Omar A, Zhang Q, Zou S, Gong J. Morphology and Phylogeny of the Soil Ciliate *Metopus yantaiensis* n. sp. (Ciliophora, Metopida), with Identification of the Intracellular Bacteria. *J Eukaryot Microbiol.* 2017; **64**:792–805. <https://doi.org/10.1111/jeu.12411>

Beinart R, Rotterová J. Starvation & washing protocol for anaerobic ciliates. 2019. Protocols.io <https://dx.doi.org/10.17504/protocols.io.868hzhw>

Beinart RA, Rotterová J, Čepička I, Gast RJ, Edgcomb VP. The genome of an endosymbiotic methanogen is very similar to those of its free‐living relatives. *Environ Microbiol.* 2018; **20**:2538–2551. <https://doi.org/10.1111/1462-2920.14279>

Raskin L, Stromley JM, Rittmann BE, Stahl DA. Group-specific 16S rRNA hybridization probes to describe natural communities of methanogens. Appl Environ Microbiol. 1994; 60:1232–1240. <https://doi.org/10.1128%2Faem.60.4.1232-1240.1994>

Crocetti G, Murto M, Björnsson L. An update and optimisation of oligonucleotide probes targeting methanogenic Archaea for use in fluorescence in situ hybridisation (FISH). *J Microbiol Methods.* 2006; **65**:194–201. <https://doi.org/10.1016/j.mimet.2005.07.007>

Wallner G, Amann R, Beisker W. Optimizing fluorescent in situ hybridization with rRNA‐targeted oligonucleotide probes for flow cytometric identification of microorganisms. *Cytometry.* 1993; **14**:136–143. <https://doi.org/10.1002/cyto.990140205>

Medlin L, Elwood HJ, Stickel S, Sogin ML. The characterization of enzymatically amplified eukaryotic 16S-like rRNA-coding regions. *Gene.* 1988; **71**:491–499. <https://doi.org/10.1016/0378-1119(88)90066-2>

Irbis C, Ushida K. Detection of methanogens and proteobacteria from a single cell of rumen ciliate protozoa. *J Gen Appl Microbiol.* 2004; **50**:203–212. <https://doi.org/10.2323/jgam.50.203>

Van Hoek AHAM, Van Alen TA, Sprakel VSI, Leunissen JAM, Brigge T, Vogels GD, *et al.* Multiple Acquisition of Methanogenic Archaeal Symbionts by Anaerobic Ciliates. *Mol Biol Evol.* 2000; **17**:251–258. <https://doi.org/10.1093/oxfordjournals.molbev.a026304>

Medlin L, Elwood HJ, Stickel S, Sogin ML. The characterization of enzymatically amplified eukaryotic 16S-like rRNA-coding regions. *Gene.* 1988; **71**:491–499. <https://doi.org/10.1016/0378-1119(88)90066-2>

Narihiro T, Sekiguchi Y. Oligonucleotide primers, probes and molecular methods for the environmental monitoring of methanogenic archaea. *Microb Biotechnol*. 2011; **4(5**):585-602. doi:10.1111/j.1751-7915.2010.00239.x

Katoh K. MAFFT: a novel method for rapid multiple sequence alignment based on fast Fourier transform. *Nucleic Acids Res.* 2002; **30**:3059–3066. <https://doi.org/10.1093/nar/gkf436>

Larsson A. AliView: a fast and lightweight alignment viewer and editor for large datasets. *Bioinformatics.* 2014; **30**:3276–3278. <https://doi.org/10.1093/bioinformatics/btu531>

Pruesse E, Peplies J, Glöckner FO. SINA: Accurate high-throughput multiple sequence alignment of ribosomal RNA genes. *Bioinformatics.* 2012; **28**:1823–1829. <https://doi.org/10.1093/bioinformatics/bts252>

1. **Supplemental tables**

**Table S1.** Origin of the ciliate strains used in this study.

**Table S2.** Sample preparation.

**Table S3.** Sample metadata utilized during analyses in PHYLOSEQ.

**Table S4.** Absolute count table with contaminants removed and ASVs renamed as described in the Methods.

**Table S5.** Relative abundance count table with contaminants removed and ASVs renamed as described in the Methods.

**Table S6.** Taxonomic assignments of ASVs retained after contaminant removal, with ASVs renamed as described in the Methods.

**Table S7.** Genetic distance matrix of the identified symbiotic methanogen lineages and the identified dominant ASVs.

**Table S8.** Percentage identity of the identified symbiotic methanogen lineages and related sequences from GenBank for Methanobacteriales.

**Table S9.** Percentage identity of the identified symbiotic methanogen lineages and related sequences from GenBank for Methanomicrobiales.

**Table S10.** Detection of methanogenic symbionts through methanogen-specific autofluorescence F420 coenzyme within the cells of the studied ciliate strains.

**Tables S11.** Sample preparation of the control medium of the examined cultures.

**Table S12.** Uncorrected genetic distances among all the obtained Sanger sequences of the methanogenic symbionts originated from the studied ciliates.

**Table S13.** Results from full and partial Mantel tests conducted between host and symbiont genetic distances and geographic distances, as described in the Methods.

1. **Supplemental files**

SF1_ASVs.fasta

SF2_Sanger_seqs_symbionts_16S_131.fasta

SF3_Sanger_seqs_host_18S_54.fasta

SF4_Sanger_seqs_symbionts_16S_131_aln_trim.fasta

SF5_Sanger_seqs_Media_55.fasta

SF6_Dataset_host_54_phylogeny_aln.fasta

SF7_Dataset_host_54_phylogeny_aln_trim.fasta

SF8_Dataset_host_phylogeny_extended.fasta

SF9_Dataset_host_phylogeny_extended_aln.fasta

SF10_Dataset_host_phylogeny_extended_aln_trim.fasta

SF11_Dataset_Methanobacteriales_uniquelineages.fasta

SF12_Dataset_Methanobacteriales_uniquelineages_aln.fasta

SF13_Dataset_Methanobacteriales_uniquelineages_aln_trim.fasta

SF14_Dataset_Methanobacteriales_all.fasta

SF15_Dataset_Methanobacteriales_all_aln.fasta

SF16_Dataset_Methanobacteriales_all_aln_trim.fasta

SF17_Dataset_Methanomicrobiales_uniquelineages.fasta

SF18_Dataset_Methanomicrobiales_uniquelineages_aln.fasta

SF19_Dataset_Methanomicrobiales_uniquelineages_aln_trim.fasta

SF20_Dataset_Methanomicrobiales_all.fasta

SF21_Dataset_Methanomicrobiales_all_aln.fasta

SF22_Dataset_Methanomicrobiales_all_aln_trim.fasta

SF23_GeneticDistance_ASVs_vs_Methanoregula_lineages.fasta

SF24_GeneticDistance_ASVs_vs_Methanoregula_lineages_aln_trim.fasta

SF25_GeneticDistance_ASVs_vs_Methanobacterium_lineages_aln_trim.fasta

SF26_GeneticDistance_ASVs_vs_Methanocorpusculum_lineages.fasta

SF27_GeneticDistance_ASVs_vs_Methanocorpusculum_lineages_aln_trim.fasta

SF28_GeneticDistance_ASVs_vs_Methanobacterium_lineages.fasta
