# Supplementary material for: Methanogenic symbionts of anaerobic ciliates are host and habitat specific
Source: ISME J. 2024 Aug 20;18(1):wrae164. doi: 10.1093/ismejo/wrae164 (PMC11378729; doi:10.1093/ismejo/wrae164)
Supplement: Supplementary_material [file supplementary_material.zip › TableS13_MantelTest.docx]

| **Table S13.** Results from full and partial Mantel tests conducted between host and symbiont genetic distances and geographic distances, as described in the Methods.  Significant values are indicated in bold. Italics indicates partial Mantel test with geography as correcting matrix. | | | | |
| --- | --- | --- | --- | --- |
|  | Host-symbiont | *Host-symbiont-geography* | Host-geography | Symbiont-geography |
| r statistic | **0.1689** | ***0.1529*** | **0.1544** | 0.02495 |
| p value | **0.001** | ***0.001*** | **0.014** | 0.214 |
